# Healthy diets ASAP – Australian Standardised Affordability and Pricing methods protocol

**DOI:** 10.1186/s12937-018-0396-0

**Published:** 2018-09-27

**Authors:** Amanda J Lee, Sarah Kane, Meron Lewis, Elizabeth Good, Christina M Pollard, Timothy J Landrigan, Mathew Dick

**Affiliations:** 1 The Australian Prevention Partnership Centre, The Sax Institute, 10 Jones Street, Ultimo, NSW 2007 Australia; 2 School of Public Health and Social Work, Queensland University of Technology, Brisbane, QLD Australia; 3 Preventive Health Branch, Department of Health, Queensland Government, Brisbane, QLD Australia; 4 School of Public Health, Curtin University, Kent Street, GPO Box U1987, Perth, 6845 Western Australia; 5 Public Health Division, Department of Health, Government of Western Australia, 189 Royal Street, East Perth, 6004 Western Australia

**Keywords:** Diet price, Food price, Diet affordability, Food affordability, Food policy, Food environments, Healthy diets, INFORMAS, Fiscal policy, Nutrition policy, Obesity prevention, Non-communicable disease, Monitoring and surveillance

## Abstract

**Background:**

This paper describes the rationale, development and final protocol of the Healthy Diets Australian Standardised Affordability and Pricing (ASAP) method which aims to assess, compare and monitor the price, price differential and affordability of healthy (recommended) and current (unhealthy) diets in Australia. The protocol is consistent with the International Network for Food and Obesity / non-communicable Diseases Research, Monitoring and Action Support’s (INFORMAS) optimal approach to monitor food price and affordability globally.

**Methods:**

The Healthy Diets ASAP protocol was developed based on literature review, drafting, piloting and revising, with key stakeholder consultation at all stages, including at a national forum.

**Discussion:**

The protocol was developed in five parts. Firstly, for the healthy (recommended) and current (unhealthy) diet pricing tools; secondly for calculation of median and low-income household incomes; thirdly for store location and sampling; fourthly for price data collection, and; finally for analysis and reporting. The Healthy Diets ASAP protocol constitutes a standardised approach to assess diet price and affordability to inform development of nutrition policy actions to reduce rates of diet-related chronic disease in Australia. It demonstrates application of the INFORMAS optimum food price and affordability methods at country level. Its wide application would enhance monitoring and utility of dietary price and affordability data from a health perspective in Australia. The protocol could be adapted in other countries to monitor the price, price differential and affordability of current and healthy diets.

**Electronic supplementary material:**

The online version of this article (10.1186/s12937-018-0396-0) contains supplementary material, which is available to authorized users.

## Background

Poor diet is now the major preventable disease risk factor contributing to burden of disease, globally and in Australia [1]. Less than 4 % of the population consume diets consistent with the evidence-based Australian Dietary Guidelines [2, 3]; on average, at least 35% of the total daily energy intake of adults and at least 39% of the energy intake of children [4] are now derived from unhealthy ‘discretionary’ food choices, defined as foods and drinks high in saturated fat, added sugar, salt and/or alcohol that are not required for health [3]. Of particular concern is the contribution of poor diet to the rising rates of overweight and obesity. Based on measured height and weight, 25% of Australian children aged two to 17 years and 63% of Australian adults aged 18 years and over are now overweight or obese [5]. There is an urgent need for nutrition policy actions to help shift the current diet of the population towards healthy diets as recommended by the Australian Dietary Guidelines [3, 6].

The expense of healthy foods has been reported as a key barrier to consumption in Australia, particularly among low socioeconomic groups [7–11]. However, well-defined data in this area are lacking [6] as classification of ‘healthy’ and ‘unhealthy’ foods and diets varies [12, 13] and the relative price of ‘healthy’ and ‘unhealthy’ foods depends on the unit of measure (i.e. per energy unit, nutrient density, serve or weight) [14]. Comparisons can be difficult particularly in the context of the total diet and habitual dietary patterns that are the major determinant of diet-related disease [3, 15–17]. However, the relative price and affordability of current and healthy (recommended) *diets* have been assessed rarely, as opposed to the relative price of selected pairs of ‘healthy’ and ‘less healthy’ foods [6].

Various methods have been utilised to assess food prices in Australia, such as Consumer Price Indexes (CPI) [18, 19] and supermarket price surveys, however these usually tally the price of highly selected individual food items and do not necessarily relate to relative cost of the total habitual diet [6, 13]. A variety of ‘food basket’ diet costing tools have also been developed at state, regional and community levels [13]. These methods have the potential to measure the cost of a healthy diet. However, dissimilarity of metrics is a recognised barrier to the production of comparable data [7, 13].

A recent systematic review of food pricing methods used in Australia since 1995, identified 59 discrete surveys using five major food basket pricing tools (used in multiple survey areas and multiple time periods) and six minor food basket pricing tools (used in a single survey area or time period) [13]. No national survey had been conducted. Survey methods differed in several metrics including: type and number of foods surveyed; application of availability and/or quality measures; definition of reference households; calculation of household income; store sampling frameworks; data collection; and analysis. Hence results are not comparable across different locations or different times [13]. With exception of Queensland Health’s Healthy Food Access Basket tool revised in 2015, [20] none of these fully align with a healthy diet as recommended by the Australian Dietary Guidelines [3]. Further, none accurately reflect current Australian diets [2, 4, 5, 13].

Since 1995, the vast majority of ‘healthy’ food pricing surveys in Australia have confirmed that: food prices in rural and remote areas are up to 40% higher than those in capital cities; lower socioeconomic households need to spend a higher proportion of their income to procure healthy diets than other Australians, and food prices generally increase over time [13, 21]. Related calls for interventions, such as for freight subsidies or food subsidies for low income groups in specific regions have gone unheeded [22, 23]. Hence, it could be asserted that these surveys have had limited utility in informing fiscal and health policy [13]. As a result, there have been several calls for the development of standardised, healthy food and diet pricing survey methods nationally in Australia [24, 25] and globally [6]. There is also a need for policy-relevant data [6, 26].

The aim of relevant nutrition policy actions is to help shift the current intake of the whole population to a healthier diet consistent with dietary recommendations. Governments can manipulate food prices through a range of complex policy approaches [6]. Three common strategies to increase the affordability of ‘healthy’ foods are:

- taxing ‘unhealthy foods’ (“fat taxes”) e.g. on sugar sweetened beverages;
- exempting ‘healthy foods’ from goods and service tax (GST) or value added tax; and
- subsidising ‘healthy foods,’ such as through agricultural and transport subsidies, retail price reductions, or voucher systems targeted to vulnerable population groups [6].

Therefore, to inform relevant policy decisions, robust data are required for both current (unhealthy) and healthy (recommended) diets [6]. With respect to food price and affordability, the key health and nutrition policy relevant question to be answered by food pricing surveys is: “What is the relative price and affordability of ‘current’ (unhealthy) and ‘healthy’ (recommended) diets?”

While the potential effects of specific changes to fiscal policy have been modelled [27, 28], recent ‘real life’ data are lacking to inform policy decision making in Australia [29]. Assessment of the price, price differential and affordability of a healthy diet (consistent with Dietary Guidelines) and current (unhealthy) diets (based on national surveys), determined by standardised national methods, would provide more robust data to inform health and fiscal policy in Australia and monitor potential fiscal policy interventions [13].

There is a lack of such data globally; the current research helps to address this, within the food price module of the International Network for Food and Obesity/non-communicable diseases Research, Monitoring and Action Support (INFORMAS) [6, 30]. Under the auspices of INFORMAS, the results of this study provide a potential globally-applicable stepwise food price and affordability monitoring framework that advocates ‘minimal’, ‘expanded’ and ‘optimal’ approaches, to establish benchmarks and monitor the cost of healthy food, meals and diets; the level depends on availability of data and country capacity [6]. The novel INFORMAS ‘optimal’ approach proposes concurrent application of two food pricing tools to assess the price, price differential and affordability of a healthy diet (consistent with Dietary Guidelines) and current (unhealthy) diets (based on national surveys). It requires assessment of household income, representative sampling and, ideally, stratification by region and socio-economic status (SES).

Based on the ‘optimal’ approach of the INFORMAS diet price and affordability framework, we developed a standardised method to assess and compare the price and affordability of healthy and current diets in Australia, provide more robust, meaningful data to inform health and fiscal policy in Australia, and develop national data benchmarks with the potential for international comparisons [29].

This paper presents the resultant protocol for Healthy Diets ASAP methods in Australia.

## Aim

The aim of this paper is to describe the development and final protocol of the Healthy Diets ASAP methods, based on the INFORMAS optimal price and affordability approach. It details tools and methods to assist others to apply the approach in a standard manner, in order to enable comparison of the price, price differential and affordability of healthy (recommended) and current (unhealthy) diets in Australia.

## Methods

### Development of the healthy diets ASAP protocol

#### Background: Developing and piloting the initial diet pricing tools and methods

In November 2013, all key Australian stakeholders gave in-principle support at a national teleconference for the development of national food price and affordability monitoring methods based on the INFORMAS ‘optimal’ approach. The development and pilot testing of the methods using readily available dietary data for five household structures in high socio-economic (SES) and low SES areas is reported elsewhere [29]. The findings confirmed that the general approach could provide useful, meaningful data to inform potential fiscal and health policy actions. Application of the diet pricing tools accurately reflected known composite food group ratios [2] and the proportion of the mean food budget Australian households spent on discretionary foods and drinks in analysis of the Consumer Price Index (CPI) with respect to Australian Dietary Guidelines food groups [19]. However, internal validity testing suggested that construction of some of the initial diet pricing tools could be improved to enhance accuracy [29]. For example, while performance of both diet pricing tools was acceptable at household level, only the healthy diet pricing tool was acceptable at an individual level for all demographics in the sample; the unhealthy (current) diet pricing tool could be improved for the 14 year old boy and both genders aged 70 years or over [29]. Further, potential systematic errors could be minimised by the utilisation of detailed dietary survey data in the Confidentialised Unit Record Files (CURFs) from the Australian Health Survey (AHS) 2011–12 [31] and the Australian 2011–13 food composition database [32], both of which were unavailable at the time of the pilot study [29].

Development of accepted, standardised diet pricing methods also required agreement from all key stakeholders on the final approach, including accord on systematic arbitrary decisions points around application of the tools (such as whether to record the price of the next largest or smallest packet if a particular size of food was unavailable in-store). There was also a desire to simplify methods to optimise uptake and utility.

#### Development and testing of diet pricing tools and process protocols

The final Healthy Diets ASAP protocols were developed in two phases.

##### Phase one: Revising and re-testing initial tools and methods

The food pricing tools were revised based on the pilot outcomes [29] and feedback from international food pricing experts (including at the Food Pricing Workshop convened by authors AL and CP at the 14th International Society of Behavioural Nutrition and Physical Activity (ISBNPA) conference in Edinburgh May 2015).

The revised unhealthy (current) diet pricing tools reflected dietary data at the five-digit level by age and gender groupings [4] in the CURFs of the AHS 2011–12 [31]. The most commonly available branded items and unit sizes in Australian supermarkets were identified from the pilot [29]. Other minor changes, and the reasons for these, are included in Table 1.

**Table 1:** Minor revisions to the initial diet pricing tools and methods

Improvement	Aim/rational/comment
Added bottled water, olive oil, and relatively healthy pre-made “convenience” foods, such as sandwiches and cooked chicken, to the healthy (recommended) diet pricing tools	To enhance comparability with the current (unhealthy) diet pricing tools, that include comparable, but less healthy, options
Further aggregated nutritionally similar products with similar utility in both diet pricing tools (for example, ‘cabana’ and ‘bratwurst’ were grouped with ‘sausages’)	To minimize the number of items to be priced in-store to reduce survey burden and cost
Included the same food groupings in the healthy food component of both current and healthy diet pricing tools	To simplify data collection, comparison between current and healthy diets and interpretation of results
Adjusted the diet of the 8 year old girl (who was the oldest in her age/gender group) from the base Foundation Diets levels, according to the prescribed methods of Total Diet modelling to inform the 2013 revision of the Australian Guide to Healthy Eating of the Australian Dietary Guidelines [33]	To ensure adequate energy content of the constructed healthy (recommended) diet of the 8 year old girl in the reference household
Adjusted median household income at Statistical Area Level 2 (SA2) level by relevant wage price index; clarified that available data sets at SA2 level provide median gross (i.e. not disposable) household income	To incorporate the effect of inflation. Median household income at sub-national (area) level is readily available from published government sources, so has been used frequently in calculation of food affordability in Australia [13]. However, published median household income data at area level reflects gross (total) income and has not been adjusted for essential expenditures such as taxation, to reflect disposable household income; results should be interpreted accordingly.
Included a third option for estimating median disposable household income at the national level, for use in future national diet price and affordability surveys.	To enhance comparability with low (minimum) disposable income household income, that is also calculated at the national level. Median disposable household income is available only at national level currently; however data may be available at state/territory level in the future.

The revised Healthy Diets ASAP diet pricing tools and methods were applied to assess the price, price differential and affordability of current and healthy diets in six randomly selected locations in two major cities (Sydney, New South Wales and Canberra, Australian Capital Territory) in November and December 2015. The preliminary reports of these studies were provided to NSW Health and ACT Health in early 2016. Colleagues in these government departments provided feedback on the revised methods early March 2016.

##### Phase 2: Development of the final protocol

At the national Healthy Diets ASAP Methods Forum (the Forum) held in Brisbane on 10 March 2016, 25 expert stakeholders from academia, government jurisdictions and non-government organisations (see Acknowledgements) worked together to finalise the Healthy Diets ASAP tools and methods for national application in Australia. De-identified preliminary data from and feedback on the reports provided to NSW Health and ACT Health were used to highlight methodological challenges and arbitrary decision points during the Forum.

Generally, the revised tools and methods applied in Sydney and Canberra were confirmed at the Forum. However, some simplifications around arbitrary decision points were recommended (Table 2).

**Table 2:** Arbitrary decisions made by key stakeholders at the national Healthy Diets ASAP Forum

Decision Point	Forum decision- standard protocol	Rationale/other comments
Household structure		
1. Number of household structures for which results are reported? (5 different structures were developed in the pilot study)	• Report and compare results for one household structure only	• Simpler to interpret and communicate results for only one (common) household structure. Less analysis, and therefore resources, required to access diet prices, therefore the protocol is more likely to be used
2. Composition of household structure?	• 2 adults and 2 children: -adult male 31–50 yrs. old -adult female 31–50 yrs. old -boy 14 yrs. old -girl 8 yrs. old • Publish quantities of food to be included for a range of individuals (age/gender), in addition to those to be included in the selected household structure	• Most commonly used household structures in Australian studies are 6 and 4 person households • Of these household structures, use 4 as it is closest to the median Australian household size of 3 persons • Those interested in reporting results for other household structures (e.g. single parent or pensioners) could perform additional analysis post data collection
Data collection		
3. Which products should be included? a) Branded? b) Generic? c) Cheapest? d) Sales items? e) Bulk deals? f) How should any optional data collected be identified on the data collection forms?	a) Include most common market share branded products (Australia wide) b) Include generic products only if branded items are not available (but exclude ALDI supermarket which tend to stock generic products). However, consider supporting optional inclusion of cheapest generic item, including the special/sale price (also applies to inclusion of ALDI) c) Don’t specifically seek to include cheapest item. However, consider supporting optional inclusion of cheapest item, including the special/sale price (also applies to inclusion of ALDI) d) Exclude sales items (as above) e) Exclude bulk deals (i.e. two for the price of one deals) Consider adding tick box in end column of data collection form to record if costing generic/special/sale price items as optional extras	a) Include the most popular items reported in the Australian Health Survey (AHS) 2011–13 as current diet b) Inclusion of generic items has potential to bias, affect comparability and distort results over time- but could be included ifconsumption data continues to suggestincreasing intake. c) Cheapest price could also be collected to answer an optional additional question, but inclusion of cheapest price, including of sales or generic items, has potential to bias, affect comparability and distort results over time. d) As above e) As above. If optionally, collecting the cheapest price, could use multi buy price by dividing to obtain single price f) May need to use multiple data collection forms for each store or add additional data collection column if collecting optional prices
4: Unhealthy (current) diet pricing tool		
a) Adjust for known under-reporting in AHS 2011–12?	No adjustment; report as ‘best case scenario’	There are no robust data on which to base adjustment factor, so could introduce error. Analysis is not adjusted for any other reasons.
b) Confirm coding for five food group and discretionary foods?	• Tinned meat and vegetables- code as ½ veg and ½ meat • Tinned fruit – code as fruit • Ham salad sandwich- (replace with chicken salad sandwich) and code as 1/3 bread, 1/3 veg, 1/3 chicken meat • Choc-chip Muesli bar – code as discretionary • Flavoured milk – code as non-discretionary (decision consistent with ABS classification) • Processed meats (e.g. ham) – code as discretionary • Water – include ½ reported water intake as bottled water (costed) and ½ as tap water (not costed)	• Decisions should be consistent with coding used by the ABS in the AHS 2011–12 • Revisit decisions reassessed when the Australian Dietary Guidelines (ADGs) are reviewed (i.e. in 5 years’ time)
5. Healthy (recommended) diet pricing tool		
Should any extra healthy foods be included? Such as more convenience options, bottled water? Is the healthy diet unrealistic without inclusion of some discretionary foods or drinks, such as alcohol?	• Water – include ½ reported water intake as bottled water • Convenience items- confirmed inclusion of roasted chicken and sandwich– no further inclusions • Alcohol – do not include	Use the ADG Modelled Foundation diets based on rationale that: −63% Australian adults are overweight/obese -There was no adjustment for underreporting in current diet -Most Australians are not expending enough energy to allow for additional energy intake from any discretionary foods or drinks - The healthy diet should be aspirational, and reflect that associated with optimal health outcomes
6: Income data		
Should mean or median income be used? What assumptions should be used to determine indicative low income?	• Include both median household (HH) income from published data and calculated low (minimum) disposable income household (HH) income (confirmed assumptions used in pilot calculations) • Also consider reporting results against the Australian poverty line	• Median HH income is specific to location, but is pre-tax i.e. not disposable income • Low income HH calculation is not specific to location apart from rent (which is set low so rarely changes) • Poverty line is lower than 50% of the Australian median HH income • Median household income and indicative low (minimum) vs disposable household income are not comparable
7: Sampling framework		
Sampling frameworks: which areas, stores, distances (e.g. 7 km radius of centre of SA2 area) should be included?	• Sampling approach SA2 stratified by Index of Relative Socio-Economic Disadvantage for Areas (SEIFA) and including all stores within a specific radius confirmed (ALDI excluded in initial methods as above) • Requested further work to determine calculating distance away from centre for inclusion of stores	• Methods of randomisation trialled is appropriate and feasible • 7 km radius of inclusion may not be appropriate for all locations, particularly in rural areas
8: Data collection protocols		
Prioritisation of sizes and branding for pricing, as proposed on data collection sheet	• Proposed detailed methods confirmed e.g. size prescribed but if not available take next larger size first	• Detailed methods proposed are appropriate • Reflect common current practice in most locations; clear and concise; easy to follow
9: Definition of affordability		
Should affordability level be set at 25% or 30% of disposable HH income?	• May need to assess both (post hoc) but initially use 30% pending further review of the literature and international consultation	• Based on most commonly used definition in international literature from high incomecountries

The revised tools and methods were finalised according to the recommendations from the Forum. The resultant Healthy Diets ASAP protocol is described in detail in the results.

Following the Forum, the food price data collected in Sydney and Canberra in late 2015 were reanalysed according to the Healthy Diets ASAP protocol and the preliminary reports to NSW Health and ACT Health were finalised in May 2016.

## Results

### The healthy diets ASAP protocol

There are five parts to the Healthy Diets ASAP protocol.

#### The healthy diets ASAP protocol part one: Construct of the diet pricing tools

There are two diet pricing survey tools: the current (unhealthy) diet pricing tool; and the healthy (recommended) diet pricing tool (Table 3). The diet pricing survey tools include provision of quantities of food for a reference household consisting of four people, including an adult male 31–50 years old, an adult female 31–50 years old, a 14 year old boy and an 8 year old girl. An allowance for edible portion/as cooked, as specified in AUSNUT 2011–13 [32], is included in both diet pricing tools. Any post plate wastage was not estimated or included.

**Table 3:** Composition of the current (unhealthy) and healthy (recommended) diets for the reference household^a^ per fortnight

Food or drink	Quantity
Current (unhealthy) diet	
*Bottled water, still (ml)*	5296
*Artificially sweetened ‘diet’ soft drink*	2391
*Fruit*	
Apples, red, loose (g)	3497
Bananas, Cavendish, loose (g)	899
Oranges, loose (g)	1664
Fruit salad, canned in juice (g)	2046
Fruit juice	3026
*Vegetables*	
Potato, white, loose (g)	1460
Sweetcorn, canned, no added salt (g)	206
Broccoli, loose (g)	422
White cabbage, loose (g)	235
Iceberg lettuce, whole (g)	795
Carrot, loose (g)	753
Pumpkin (g)	240
Four bean mix, canned (g)	74
Diced tomatoes, canned, in tomato juice(g)	234
Onion, brown, loose (g)	84
Tomatoes, loose (g)	488
Frozen mixed vegetables, pre-packaged (g)	1184
Frozen peas, pre-packaged (g)	273
Baked beans, canned (g)	369
Salad vegs in sandwich	120
Veg in tinned meat and vegetable casserole (g)	646
*Grain (cereal) foods*	
Wholegrain cereal biscuits Weet-bix™ (g)	430
Wholemeal bread, pre-packaged (g)	1054
Rolled oats, whole (g)	870
White bread, pre-packaged (g)	3033
Cornflakes (g)	680
White pasta, spaghetti (g)	1326
White rice, medium grain (g)	1622
Dry water cracker biscuit (g)	258
Bread in sandwich	120
*Meats, poultry, fish, eggs, nuts and seeds*	
Beef mince, lean (g)	267
Lamb loin chops (g)	257
Beef rump steak (g)	1056
Tuna, canned in vegetable oil (g)	1052
Whole barbeque chicken, cooked (g)	1661
Eggs (g)	872
Meat in tinned meat and vegetable casserole (g)	646
Chicken in sandwiches	120
*Milk, yoghurt, cheese and alternatives*	
Cheddar cheese, full fat (g)	624
Cheddar cheese, reduced fat (g)	44
Milk, full fat (ml)	5961
Milk, reduced fat (ml)	2929
Yoghurt, full fat plain (g)	204
Yoghurt, reduced fat, flavoured (vanilla) (g)	676
Flavoured milk (ml)	2416
Canola margarine (g)	170
Sunflower oil (ml)	7
Olive oil (ml)	7
*Discretionary choices*	
Beer, full strength (ml)	4661
White wine, sparkling (ml)	863
Whisky (ml)	266
Red wine (ml)	1078
Butter (g)	280
Muffin, commercial (g)	1455
Cream-filled sweet biscuit, pre-packaged (g)	496
Muesli bar, pre-packaged (g)	373
Peanuts, salted (g)	255
Pizza, commercial (g)	1182
Savoury flavoured biscuits (g)	222
Confectionary (g)	418
Chocolate (g)	441
Sugar sweetened beverages (Coca Cola) (ml)	12,012
Meat pie, commercial (g)	1638
Frozen lasagne, pre-packaged (g)	4322
Hamburger, commercial (g)	2413
Beef sausages (g)	1048
Ham (g)	189
Potato crisps, pre-packaged (g)	518
Potato chips, hot, commercial (g)	670
Ice cream (g)	1830
White sugar (g)	564
Salad dressing (ml)	277
Tomato sauce (ml)	569
Chicken soup, canned (g)	1340
Orange juice (ml)	3027
Fish fillet crumbed, pre-packaged (g)	302
Instant noodles, wheat based (g)	381
Healthy (recommended) diet	
*Bottled water, still (ml)*	5296
*Fruit*	
Apples, red, loose (g)	5460
Bananas, Cavendish, loose (g)	5460
Oranges, loose (g)	5460
*Vegetables*	
Potato, white, loose (g)	2320
Sweetcorn, canned, no added salt (g)	1160
Broccoli, loose (g)	1470
White cabbage, loose (g)	1470
Iceberg lettuce, whole (g)	1470
Carrot, loose (g)	2205
Pumpkin (g)	2205
Four bean mix, canned (g)	1005
Diced tomatoes, canned, in tomato juice(g)	1638
Onion, brown, loose (g)	1638
Tomatoes, loose (g)	1638
Frozen mixed vegetables, pre-packaged (g)	1638
Frozen peas, pre-packaged (g)	1638
Baked beans, canned (g)	1005
Salad vegs in sandwich	120
*Grain (cereal) foods*	
Wholegrain cereal biscuits Weet-bix™ (g)	2216
Wholemeal bread, pre-packaged (g)	4272
Rolled oats, whole (g)	6648
White bread, pre-packaged (g)	893
Cornflakes (g)	670
White pasta, spaghetti (g)	2042
White rice, medium grain (g)	2042
Dry water cracker biscuit (g)	781
Bread in sandwich	120
*Meats, poultry, fish, eggs, nuts and seeds*	
Beef mince, lean (g)	1168
Lamb loin chops (g)	1169
Beef rump steak (g)	1172
Tuna, canned in vegetable oil (g)	1841
Whole barbeque chicken, cooked (g)	1471
Eggs (g)	2208
Peanuts, roasted, unsalted (g)	780
Chicken in sandwiches	120
*Milk, yoghurt, cheese and alternatives*	
Cheddar cheese, full fat (g)	704
Cheddar cheese, reduced fat (g)	516
Milk, full cream (ml)	6438
Milk, reduced fat (ml)	12,000
Yoghurt, full fat plain (g)	2576
Yoghurt, reduced fat, flavoured (vanilla) (g)	5100
Canola margarine (g)	412
Sunflower oil (ml)	291
Olive oil (ml)	291

^a^The reference household comprises four people: adult male 19–50 yrs. old; adult female 19–50 yrs. old; boy 14 yrs. old; girl 8 yrs. old

##### The healthy diets ASAP current (unhealthy) diet pricing tool

The current (unhealthy) diet pricing tool constitutes the sum of the mean intake of specific foods and drinks, expressed in grams or millilitres, in each age/gender group corresponding to the four individuals comprising the reference household, as reported in the AHS 2011–12 [31]. Foods are grouped according to stakeholder recommendations (Table 2) and amounts consumed per day are derived from the CURFs at 5-digit code level [31]. The mean reported daily intake for each of the four individuals (Additional file 1) are multiplied by 14 and tallied to produce the quantities consumed per household per fortnight. The amounts of foods and drinks comprising the Healthy Diets ASAP current (unhealthy) diet for the reference household per fortnight is presented in Table 3. The total energy content of the reference household’s current diet is 33,860 kJ per day. Common brands of included food and drink items are included in the data collection sheet in Table 4.

**Table 4:**
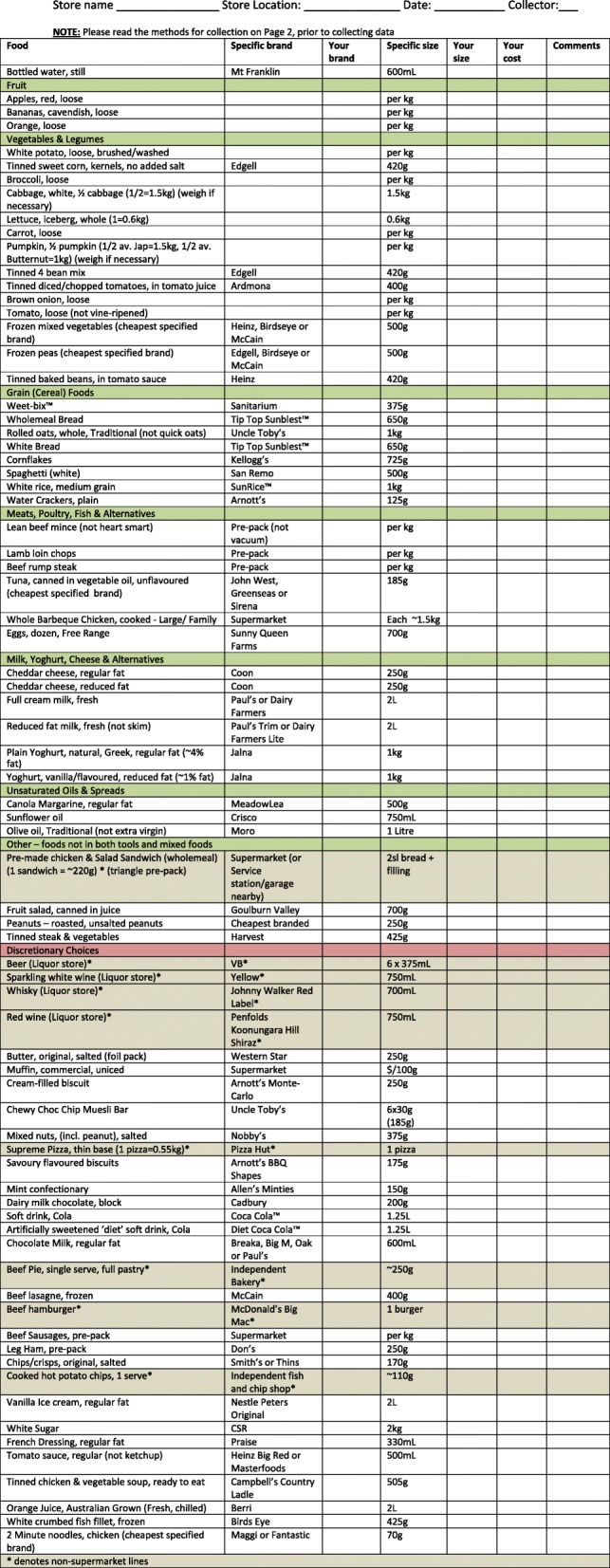
Healthy Diets ASAP (Australian Standardised Affordability and Price) Survey Data Collection Form

##### The healthy diets ASAP healthy (recommended) diet pricing tool

The healthy diet pricing tool reflects the recommended amounts and types of foods and drinks for the reference household for a fortnight, consistent with the Australian Guide to Healthy Eating and the Australian Dietary Guidelines [3]. The amounts are calculated from the daily recommended number of servings and relevant serve size of foods for the age/gender and physical activity level (PAL) of 1.5 of the four individuals comprising the reference household in the omnivorous Foundation Diet models [33]. As the Foundation Diets were developed for the smallest adults (or in the case of children, the youngest) in each age/gender group, the amounts of foods were increased by 20% for the 8 year old girl who is the oldest in her height/age group, according to the recommendations [3].

To ensure the most commonly consumed healthy foods in Australia are used, food categories in the healthy diet pricing tool are the same as those in the current diet pricing tool (but differ in quantity). A variety of fresh, canned, frozen and dried foods is included. For example, representative categories of fresh produce reflect common fruit and vegetables available all year round in Australia. Luxury products, such as imported fruit and vegetables (particularly those out of season) and foods with very high cost per kilogram (e.g. oysters, smoked salmon) are excluded. Some ‘convenience’ foods are included in the healthy diet pricing tool as per stakeholder decisions (Table 2).

Consistent with Australian recommendations [3], the healthy diet pricing tool does not contain any discretionary choices. It includes: grain (cereal) foods, in the ratio 66% wholegrain and 33% refined varieties; cheese, milk, yoghurt and calcium-fortified plant based alternatives, mostly (i.e. > 50%) reduced fat, with a maximum of 2–3 serves of high fat dairy foods (cheese) per person per week; lean meat (beef, lamb, veal, pork), poultry and plant-based alternatives (with no more than 455 g red meat per person per week); a minimum of 140 g and up to 280 g fish per person per week; up to 7 eggs per person per week; a selection of different colours and varieties of vegetables (green and brassica, orange, legumes, starchy vegetables, other vegetables) with a minimum 350 g per day for adults; a variety of fruit with a minimum of 300 g per day for adults; and an allowance of unsaturated oils or spreads or the nuts/seeds from which they are derived [33]. The daily quantities of food categories recommended for each individual (age/gender) in the reference household (Additional file 2) are multiplied by 14 and tallied to provide quantities per fortnight (Table 3).

The amounts of foods and drinks comprising the Healthy Diets ASAP healthy (recommended) diet for the reference household per fortnight are presented in Table 3. The total energy content of the household’s healthy diet is 33,610 kJ per day. Common brands of included food and drink items are included in the data collection sheet in Table 4.

##### Diet pricing tools for additional household structures

Several stakeholders requested (Table 2) that the composition of current (unhealthy) and healthy (recommended) diets be provided for four other household compositions commonly investigated in Australia^1^ (for example, for single parent or pensioner households) so that additional data analysis could be performed. These data are included in Additional file 3.

##### Validity of the diet pricing survey tools

Convergent validity of the constructed healthy and current diet pricing survey tools for each age/gender group was assessed by energy and macronutrient analysis using FoodWorks 7 Professional [34] computer program installed with AUSNUT 2011–13 [32] (the food composition database used to analyse the AHS) and comparing results with Australian Nutrient Reference Values [35] and nutrient results from the AHS 2011–12 respectively [5, 31]. The results are presented in Additional file 4. As deemed acceptable for modelling outputs to develop the Australian Guide to Healthy Eating, [33] the energy content of the constructed healthy diet pricing tool is within 5% of the Foundation Diet levels and the macronutrient profiles are within the recommended ranges for more than 97% of values for all age/gender groups. Similarly, the energy content of the current diet pricing tool is within 5% of the reported energy intakes of the AHS 2011–2012 [4] for all individuals.

Internal validity indicators, such as the ratio of fruit and vegetables content between the healthy and current diet pricing tools (approximately 2:1) are consistent with available published data [2, 31] and recommendations [33]. Further, the proportion of household food expenditure on discretionary items (around 58%) [29] is similar to that described by the ABS (58.2%) using different methods based on household expenditure [19]. Hence the tools appear valid for use in estimating the cost of current and healthy diets.

#### The healthy diets ASAP protocol part two: Location and store sample selection

A random sample of the Statistical Area Level 2 (SA2) locations in each town is selected to achieve a representative sample. SA2 locations are stratified by the Index of Relative Socio-Economic Disadvantage for Areas (SEIFA) quintile using information and maps available on the ABS website [36–38] Following sample size calculations, the required number of SA2 locations within SEIFA Quintile 1, 3 and 5 are selected randomly for participation. Food outlets within seven kilometres by car of the centre of each SA2 location are identified with Google™ Maps [39] and included in the surveys. Stores to survey include one outlet of all supermarket chains (in trials these were Coles™, Woolworths™ and Independent Grocers Australia (IGA™), Supabarn™ and ALDI™), ‘fast-food’/take-away outlets (a Big Mac™ hamburger from the McDonald’s™ chain; pizza from the Pizza Hut™ chain; fish and chips from independent outlets) and two alcoholic liquor outlets closest to the geographical centre of each SA2 location.

#### The healthy diets ASAP protocol part three: Collecting and entering food price data

The Healthy Diets ASAP diet price survey data collection form (Table 4) combines the items included in the current diet and the healthy diet for convenience and utility. The agreed price data collection protocol is presented in Table 5 and is printed on each data collection form. Research assistants are trained to use the form and follow the price collection protocol strictly. Prices are collected within the same 4 week/monthly period, as prices change over time.

**Table 5:** Healthy Diets ASAP food price data collection protocol

1. Record the usual price of an item, i.e. do not collect the sale/special price unless it is the only price available (if so, note in comment column) 2. Look for the specified brand and specified size for each food item, and record the price • If the specified brand is not available: Choose the cheapest brand (non-generic) available in the specified size. Note this brand in the “Your brand” column • If the specified size is not available: Choose the nearest larger size in the specified brand. If a larger size is not available, choose the nearest smaller size. Note this size in the “Your size” column • If both the specified brand and specified size are not available: Choose the cheapest in the nearest larger size of another brand (non-generic). If a larger size is not available, choose the nearest smaller size • If multiple brands are specified, record the price of the cheapest one and note brand in the “Your brand” column • If the item is only available in a generic form (e.g. Home Brand, Coles, Woolworths Select, Black and Gold) choose the most expensive generic item in the specified size. If the specified size is not available, choose the nearest larger size. If a larger size is not available, choose the nearest smaller size. Note the generic name in the “Your brand” and the size in the “Your size” columns 3. Loose produce: choose the usual cheapest price per kg of the variety not on special. If the only variety available is on special, record the special price and note in comments column 4. Peanuts: choose the branded packet size closest to 250 g. If packaged, roasted, unsalted peanuts are not available, record the price of the loose ‘bulk scoop & weigh’ roasted, unsalted peanuts per 100 g 5. Check all data are collected and recorded as above, before leaving store	

Permission to participate is sought from each store manager prior to data collection.

Data entry and analysis sheets have been developed using Excel™ spreadsheets [40]. Double data entry is recommended to minimise error. Data are cleaned and checked. Any missing values are imputed to ascribe the mean price of the same food item in all other relevant outlets in the same SA2 area. Data analysis tools are available from the corresponding author. As has been achieved previously for the Victorian Health Food Access Basket [41], the Healthy Diets ASAP App is under development to streamline data collection and analysis and reduce error.

#### The healthy diets ASAP protocol part four: Determination of household income

Household income is determined by either of three methods, depending on the purpose of the study and the granularity of available data.

##### Median household gross income at area level

In Australia, national census data is the only source of SA2 level household income data and is provided only at total (gross) level. Median gross household income is determined per week (before taxation, rent and other expenses) in each SA2 area by entering relevant post codes into the Community Profile data calculator [42] that is based on the 2011 Census results [36], adjusted for the wage price index (for example, there was an increase of 11.1% from September 2011 to September 2015) and multiplying by two to derive median household income in each SA2 area per fortnight. Details and examples are provided in Additional file 5.

##### Indicative low (minimum) disposable household income

Indicative low (minimum) income of the reference household (and other households of interest to specific stakeholders) is calculated based on the level of minimum wages [43] and determination of the welfare payments provided by the Department of Human Services [44] as per the methods used by the Queensland Department of Health [20]. Assumptions are made for employment, housing type, disability status, savings and investments, child support, education attendance and immunisation status of children (Table 6). As welfare policy actions can change, the most recent schedules should be used. Where it is higher than the minimum threshold, the indicative low (minimum) household income is adjusted for taxation payable [45] so also represents minimum household disposable income. Details and examples are provided in Additional file 6.

**Table 6:** Assumptions applied to determine the indicative low (minimum) disposable household income of the reference household

The reference household consists of an adult male, an adult female, a 14 year old boy and an 8 year old girl • The adult male works on a permanent basis at the national minimum wage ($17.29 per hour)for 38 h a week • The adult female works on a part-time basis at the national minimum wage ($17.29 per hour)for 6 h a week • Both children attend school and are fully immunised • None of the family are disabled • The family has some emergency savings that earn negligible interest	

##### Median household disposable income at national level

For assessment of diet affordability at the national level, median equivalised disposable household income for the reference family composition is sourced from t*he Survey of Income and Housing* [46]*.*

#### The healthy diets ASAP protocol part five: Data analysis and reporting

The price of the healthy (recommended) and current diets in each store and the mean price for each SEIFA quintile is calculated for the reference household composition in each of areas surveyed in each city. Results can be presented in a range of metrics, including the cost of the total diets per household per fortnight, and the cost of purchasing specific five food group and discretionary foods and drinks (including policy relevant items such as alcohol, ‘take-away foods’ and sugar-sweetened beverages). The results for the current (unhealthy) diet and healthy (recommended) diet are compared to determine the differential.

Affordability of the healthy and current diets for the reference household is determined by comparing the cost of each diet with the median gross household income (Additional file 5) and also with the indicative low (minimum) disposable income of low income households (Additional file 6). Where a representative national survey of diet prices has been conducted, affordability of the healthy and current diets for the reference household is determined by comparing the cost of each diet with the median equivalised disposable income [46] and with the indicative low (minimum) disposable income of low income households. Internationally, a benchmark of 30% of income has been used to indicate affordability of a diet [6, 10].

Data files can be manipulated to investigate the effects of potential fiscal policy changes on the affordability of current (unhealthy) and healthy (recommended) diets for the reference household. The price of the relevant foods and drinks can be modified readily to highlight the likely ‘real-world’ impacts of different scenarios, for example, to investigate the potential extension of the Goods and Services Tax (GST) on basic healthy foods [47], or the potential application of different levels of taxation on sugary drinks in Australia [29].

## Discussion

There are several methodological limitations inherent in the Healthy Diets ASAP protocols. Given that it is based on national reported mean dietary intakes, the cost of the current (unhealthy) diet is unlikely to be the same as actual expenditure on food and drinks in specific areas and among specific groups [48]. Other assumptions commonly made in similar apparent consumption and household expenditure surveys include that food is shared equitably throughout the household, that there is no home food production and minimal wastage. Nutritionally similar products were aggregated to minimise the number of items included in the diet pricing tools, but products were not necessarily homogenous in terms of price. However, similar healthy food items were included in each diet to try to minimise any unintended effects.

Ideally, the specific foods included in both diet pricing tools are culturally acceptable, commonly consumed, widely available, accessible and considered ‘every day’ rather than luxury items. As the foods and drinks included in the current diet pricing survey tool reflect actual consumption data, it was presumed that they were deemed by the population as a whole as meeting these requirements. No adjustments were made for costs such as transport, time, cooking equipment and utilities; as these apply to both current and healthy diets, assessment of the price differential between the two can help control for some of these hidden costs to some extent. However, these hidden costs would increase actual diet costs and decrease affordability of the diets.

No adjustments were made to account for the marked under-reporting in the AHS 2011–12 [4], reported dietary variability amongst different groups other than age/gender stratification, or the greater proportion of pre-prepared ‘convenience’ items in the current diet pricing tool compared with the healthy diet pricing tool. Given the high rates of overweight/obesity in Australia, the Foundation Diets were prescribed for the shortest and least active in each age group according to the modelling that informed the Australian Guide to Healthy Eating [33]; however this would under-estimate the requirements of taller, more active and healthy weight individuals.

No attempt was made to control the price of the healthy diet pricing tool or the current diet pricing tool for energy, as the diets are constructed on recommended energy levels and actual reported levels of energy respectively. Further, the energy content of each tool is a determinant variable that directly affects diet-related health outcomes [18, 49]. As most Australians are already overweight or obese, increasing recommended energy requirements in excess of Foundation Diets is not consistent with optimum health outcomes [33]. As the key exposure variable affecting the life time risk of diet-related disease is the total diet and dietary patterns, approaches such as this that compare metrics of actual current diets with recommended diets are more pertinent to the health policy debate than the more common, but limited, studies into the relative price of selected ‘healthy’ and ‘unhealthy’ foods or single ‘optimised’ diets [18, 50, 51].

While a benchmark of 30% of income has been used to indicate affordability of diet internationally and in Australia [6, 9, 10] it is not clear from the literature whether this income comparator is gross income or disposable income [6]. Using disposable income to estimate affordability better reflects the capacity of a household to afford food/diets [52, 53]; using gross income is a more conservative approach as it does not take taxation into account. However, in Australia currently, median disposable household income data are readily available only at national level [46]; at area level only median gross household income data are readily available. Further, the composition of the reference household does not align necessarily with that of households in the census in all areas. Comparing diet price with indicative low (minimum) disposable household more accurately estimates affordability of diets in vulnerable groups. However, the tax paid component of indicative low (minimum) disposable household income can be removed to improve comparability with estimates of affordability determined by application of gross median household income.

Arbitrary decision points occur around sampling frameworks, data collection protocols (for example, selection of cheapest comparable generic item if the branded item is unavailable in any size), analysis and presentation of results, data sources and definitions of family and household income and composition. Such methodological limitations are common to other food price studies. In order for final methods to be replicable, agreement among key stakeholders including end users on each of these decision points at the Healthy Diets ASAP Forum was invaluable. Publication of detailed protocols is essential to support uptake, replicability, fidelity and transparency of the method.

The detailed dietary survey data required to produce the current (unhealthy) diet pricing tool and the modelling data required to produce the healthy (recommended) diet pricing tool are not easily accessible in all countries and technical capacity to analyse individual records may be limited. Therefore, this optimal approach may be too complex for application to assess and monitor the price of diets from a health perspective globally. However, there is potential for the diet pricing tools to be adapted for use in other countries by substitution of food components with commonly-consumed local equivalents, dietary analysis and testing.

## Conclusion

The development of standardised Healthy Diets ASAP method protocols provides an example of how the INFORMAS optimal food price and affordability methods can be adapted at country level to help develop standardised, policy relevant diet price assessment, monitoring tools and benchmarks. The approach can be used to assess the price, price differential and relative affordability of current (unhealthy) and healthy (recommended) diets and inform scenario modelling of potential fiscal and nutrition policy actions.

The Healthy Diets ASAP method satisfies long-standing calls for the development of a nationally standardised approach to assess food prices from a health perspective, supporting comparison of results from different locations and over time, in Australia.

The protocol could be adapted in other countries to benchmark and monitor the price, price differential and affordability of current and healthy diets globally.

## Additional files

Additional file 1: Current (unhealthy) Diets: Mean daily intake of representative categories of foods and drinks for individuals (age/gender) comprising the reference household, and other common households. (DOCX 43 kb)

Additional file 2: A. Foundation diet recommended serves of foods per week for individuals (NHMRC 2011) comprising the reference household and other common households. B. Healthy (recommended) Diets: Recommended serves per day of food groups and amounts of composite foods and drinks for individuals comprising the reference household, consistent with Foundation Diets (NHMRC 2011) including commonly-consumed brands. (DOCX 55 kb)

Additional file 3: Composition of the current (unhealthy) diet and healthy (recommended) diet for four additional households (HH1, HH2, HH3, HH4)^1^ per fortnight. (DOC 188 kb)

Additional file 4: Energy and nutrient analysis of individual current and healthy diet baskets compared to results of the AHS and Foundation Diet modelling. (DOCX 36 kb)

Additional file 5: Median income determination by SA2 Example- Median income data from the 2011 Census, ABS Community Profiles of SA2 areas for six SA2 locations in Sydney, NSW*. (DOCX 36 kb)

Additional file 6: Calculations of low (minimum) disposable household income data from welfare data – Example. (DOCX 34 kb)

## Abbreviations

ASAP: Australian Standardised Affordability and Pricing methods
CURFs: Confidential Unit Record Files

## Footnotes

1 Additional data are provided for:

- Household 1(*n* = 6): adult male 31–50 yrs. old; adult female 31–50 yrs. old; older female 70+ yrs. old; boy 14 yrs. old; girl 8 yrs. old; boy 4 yrs. old
- Household 2 (*n* = 3): single parent with 2 children: adult female 31–50 yrs. old; boy 14 yrs. old; girl 8 yrs. old
- Household 3 (*n* = 1): single unemployed person: adult male 31–50 yrs. old
- Household 4 (*n* = 2): older couple with no children: senior adult male 70+ yrs. old; senior adult female 70+ yrs. old: pensioners
