# Supplementary material for: Healthy diets ASAP – Australian Standardised Affordability and Pricing methods protocol
Source: Nutr J. 2018 Sep 27;17:88. doi: 10.1186/s12937-018-0396-0 (PMC6161417; doi:10.1186/s12937-018-0396-0)
Supplement: Supplementary file 1 — Current (unhealthy) Diets: Mean daily intake of representative categories of foods and drinks for individuals (age/gender) comprising the reference household, and other common households. (DOCX 43 kb) [file 12937_2018_396_MOESM1_ESM.docx]

| **Additional file 1: Current (unhealthy) Diets: Mean daily intake of representative categories of foods and drinks for individuals (age/gender) comprising the reference household, and other common households** | | | |  |  |  |  |  |
| --- | --- | --- | --- | --- | --- | --- | --- | --- |
| **Food item** | **Males 31-50yo (g)** | **Females 31-50yo (g)** | **Males 71yo+ (g)** | **Females 71yo+ (g)** | **Males 14yo (g)** | **Females 8yo (g)** | **Males 4yo (g)** |  |
| **Water** |  |  |  |  |  |  |  |  |
| Water, bottled | 144.3607684 | 115.3009465 | 34.96705576 | 30.9704465 | 22.4811258 | 96.12540955 | 15.63945725 |  |
| **FRUIT** |  |  |  |  |  |  |  |  |
| Apples | 51.60551731 | 45.85693195 | 47.21378128 | 43.57663578 | 41.98006309 | 110.3237552 | 68.97289162 |  |
| Bananas | 20.94410948 | 16.93634481 | 23.70490678 | 29.34285112 | 4.066927891 | 22.24730045 | 30.80234464 |  |
| Oranges | 25.70493566 | 24.36093827 | 31.39227925 | 25.12917885 | 33.99704966 | 34.81101507 | 34.30665002 |  |
| Fruit salad, canned in juice | 44.33723228 | 52.97678529 | 78.80607423 | 65.25940149 | 26.76659675 | 22.06143088 | 61.39880648 |  |
| **VEGETABLES AND LEGUMES** |  |  |  |  |  |  |  |  |
| Potato, loose | 36.82198294 | 27.92673814 | 52.9232121 | 40.23561719 | 19.39399499 | 20.10964217 | 11.29424852 |  |
| Sweetcorn, canned | 5.474430583 | 4.856752023 | 4.54651344 | 6.957920268 | 3.505588079 | 0.898339388 | 8.784466042 |  |
| Broccoli, loose | 10.29421381 | 10.90904608 | 7.006606559 | 10.79106169 | 5.987229752 | 2.916692334 | 3.393559741 |  |
| White cabbage, loose | 6.687854264 | 5.992043242 | 7.09437544 | 5.331205896 | 2.350268171 | 1.736985558 | 1.572553777 |  |
| Iceberg lettuce, whole | 23.35301407 | 26.85071314 | 13.0869849 | 16.73524669 | 3.494516375 | 3.083886453 | 3.002712425 |  |
| Carrot, loose | 12.1495814 | 14.51835923 | 17.41572047 | 17.78063593 | 7.458675806 | 19.67704073 | 9.106768193 |  |
| Pumpkin | 6.118363349 | 8.193001144 | 8.902412662 | 11.57014283 | 0 | 2.82229238 | 0.380002916 |  |
| Four bean mix, canned | 1.714768078 | 2.238881997 | 1.752506604 | 0.800791783 | 1.241916201 | 0.123623206 | 1.813620678 |  |
| Diced tomatoes, canned | 6.670118633 | 4.541209422 | 3.704300637 | 2.182279981 | 3.231807311 | 2.295526153 | 3.097366469 |  |
| Onion, loose | 3.416332689 | 2.454182973 | 6.444498618 | 2.712150595 | 0.065905446 | 0.096238738 | 0.116206728 |  |
| Tomatoes, loose | 13.35479397 | 12.97315201 | 17.70519481 | 12.53852341 | 3.037220529 | 5.510907736 | 3.414279679 |  |
| Frozen mixed vegetables | 30.26461565 | 30.02176181 | 23.22572296 | 23.60678371 | 4.238765113 | 20.01289479 | 16.4268934 |  |
| Frozen peas | 7.54562786 | 6.166443427 | 7.608111322 | 9.096552592 | 2.680999814 | 3.082570778 | 1.325742317 |  |
| Baked Beans, canned | 9.127436662 | 7.474170553 | 10.58795274 | 6.779542174 | 4.92406313 | 4.82863508 | 4.375041591 |  |
| **Grain (Cereal) Foods – Wholegrain and Refined** | |  |  |  |  |  |  |  |
| Weetbix | 11.96284866 | 9.772623641 | 12.49904055 | 7.862845849 | 5.186910687 | 3.826389693 | 2.053837937 |  |
| Wholemeal bread, pre-packaged | 26.44805518 | 19.93977979 | 41.4926993 | 33.87571104 | 8.289716956 | 20.57886256 | 10.89083501 |  |
| Rolled oats | 18.19940822 | 16.70295183 | 29.25498762 | 37.21292936 | 9.53004671 | 17.67805291 | 17.04534164 |  |
| White bread, pre-packaged | 49.21100773 | 35.36940259 | 47.11161329 | 30.92963896 | 81.53944569 | 50.52198491 | 53.52211783 |  |
| Cornflakes | 11.08114232 | 5.855787465 | 14.83858296 | 8.779154398 | 15.13120213 | 16.48946548 | 15.47543451 |  |
| White pasta | 27.41377947 | 20.68207137 | 14.89973524 | 9.125594506 | 25.74612148 | 20.85320716 | 15.38179097 |  |
| White rice | 37.44005646 | 30.49765297 | 11.82199492 | 12.07678701 | 27.18889446 | 20.75298939 | 29.6945594 |  |
| Dry wheat crackers, water crackers | 2.341935545 | 3.430045178 | 1.70202542 | 2.692903339 | 1.268224284 | 11.36132292 | 5.233143173 |  |
| **Lean Meats and Poultry, Fish, Eggs, Nuts and Seeds** | |  |  |  |  |  |  |  |
| Beef mince, lean | 7.903338626 | 6.093694589 | 6.00417986 | 6.349295417 | 4.557770142 | 0.549465422 | 2.762600768 |  |
| Lamb loin chops | 12.45968366 | 4.900950123 | 7.429038523 | 11.31602304 | 0 | 1.009476006 | 2.72133246 |  |
| Beef rump steak | 26.66129891 | 16.26699808 | 21.09537013 | 17.99717304 | 25.9060055 | 6.601885114 | 1.995309832 |  |
| Tuna, canned in oil | 21.19619257 | 21.69889184 | 18.58328055 | 20.15404711 | 14.49952115 | 17.77948963 | 10.39380131 |  |
| Chicken, cooked whole | 46.94694957 | 37.74690684 | 30.20539399 | 27.0217106 | 18.18588793 | 15.75021429 | 20.89350993 |  |
| Eggs | 21.89242911 | 18.51722973 | 18.41078795 | 18.36640871 | 11.52056671 | 10.3533618 | 12.65673911 |  |
| Canned meat and vegetable casserole | 30.50002249 | 20.07368096 | 27.86584586 | 16.62344408 | 33.40552349 | 8.238717172 | 15.6259827 |  |
| **Milk, Yoghurt, Cheese and Alternatives** |  |  |  |  |  |  |  |  |
| Cheddar cheese, full fat | 11.20404049 | 9.324016638 | 9.590768608 | 8.292461065 | 9.878283151 | 14.15712972 | 16.83631815 |  |
| Milk, full fat | 89.93535569 | 59.4670321 | 79.46212474 | 65.29588296 | 102.3394781 | 174.0565294 | 149.3851308 |  |
| Cheddar cheese, reduced fat | 1.060435373 | 1.948812773 | 0.443872633 | 1.331159954 | 0.106656581 | 0 | 0.654372359 |  |
| Milk, reduced fat | 59.13620488 | 60.61338272 | 85.18799361 | 88.13777274 | 63.77576585 | 25.71893382 | 13.19724579 |  |
| Yoghurt, full fat, plain | 5.491158972 | 6.554805359 | 3.293222028 | 4.512436975 | 1.818120564 | 0.726034366 | 3.401831635 |  |
| Yoghurt, flavoured reduced fat | 14.93172443 | 21.17583356 | 9.992393745 | 16.66539847 | 4.412805184 | 7.74144735 | 47.07172203 |  |
| **Unsaturated Oils and Spreads (or foods from which these are derived)** | | |  |  |  |  |  |  |
| Canola margarine | 3.055145731 | 1.845293124 | 5.311699042 | 4.300847443 | 3.503213668 | 3.723498697 | 2.577913071 |  |
| Sunflower oil | 0.240198201 | 0.235614376 | 0.215209793 | 0.149341996 | 0.010488144 | 0.005966037 | 0 |  |
| Olive oil | 0.240198201 | 0.235614376 | 0.215209793 | 0.149341996 | 0.010488144 | 0.005966037 | 0 |  |
| **Pre-prepared mixed foods** |  |  |  |  |  |  |  |  |
| Sandwich, pre-made, white bread, chicken, salad, cheese | 13.26828817 | 6.06228765 | 4.784956377 | 1.20609669 | 6.202494298 | 0.263929419 | 6.514123477 |  |
| **Discretionary Choices** |  |  |  |  |  |  |  |  |
| Beer, full strength | 302.320945 | 30.58065394 | 144.1510719 | 17.44856905 | 0 | 0 | 0 |  |
| White wine, sparkling | 20.76929083 | 40.8797195 | 28.04180857 | 36.72241213 | 0 | 0 | 0 |  |
| Whisky | 12.10835038 | 6.905430461 | 5.429392008 | 6.440962837 | 0 | 0 | 0 |  |
| Red wine | 41.94981553 | 35.05659803 | 47.21534521 | 21.92241085 | 0 | 0 | 0 |  |
| Butter | 4.016598051 | 3.393687904 | 7.767923818 | 5.666161438 | 11.55105337 | 1.059514809 | 6.782582442 |  |
| Muffin, commercial | 25.10796732 | 24.67150783 | 32.13963386 | 24.47012215 | 33.79588519 | 20.36181234 | 23.36042897 |  |
| Cream-filled sweet biscuit, pre-packaged | 9.753596141 | 8.157727414 | 14.70162348 | 14.60241552 | 3.071784923 | 14.44731887 | 7.613216373 |  |
| Muesli bar, pre-packaged | 5.142038996 | 3.215664152 | 1.886568587 | 1.369311316 | 10.76493555 | 7.531350726 | 7.334420807 |  |
| Nuts, mixed, salted | 7.261446782 | 6.51509661 | 5.347421455 | 3.852787019 | 3.660154114 | 0.807099195 | 1.91400838 |  |
| Pizza, commercial | 22.14920134 | 13.36155535 | 7.465472264 | 4.570979027 | 34.0306096 | 14.90337413 | 14.40724534 |  |
| Savoury flavoured biscuits | 2.844493489 | 1.815168037 | 2.699858569 | 1.439289201 | 7.938582908 | 3.230851599 | 1.448974339 |  |
| Confectionary | 3.26855179 | 5.832005801 | 4.802932374 | 3.989604641 | 13.69158052 | 7.091311426 | 10.74603498 |  |
| Chocolate | 7.699294887 | 6.857057459 | 4.285255827 | 3.523600452 | 6.892929414 | 10.01670404 | 2.714002949 |  |
| Sugar-sweetened beverages (Coca Cola) | 242.851382 | 121.741991 | 65.58652867 | 44.07943286 | 391.707318 | 101.6924863 | 63.36983517 |  |
| Artificially sweetened beverages | 69.42053488 | 62.22876739 | 28.37458449 | 8.963922506 | 37.19123282 | 1.913978749 | 10.27498567 |  |
| Flavoured milk | 63.40481074 | 31.18339637 | 25.2627305 | 17.41253768 | 48.38130598 | 29.62409809 | 36.33479777 |  |
| Meat pie, commercial | 31.68020445 | 21.65332684 | 22.41375802 | 8.960873225 | 39.05849982 | 24.59191304 | 15.64430487 |  |
| Frozen lasagne, pre-packaged | 73.12249084 | 64.12792387 | 28.43154137 | 27.79613901 | 125.5494391 | 45.88323211 | 46.99816283 |  |
| Hamburger, commercial | 47.39349984 | 25.28255107 | 14.16074995 | 11.01361853 | 67.42382414 | 32.22877417 | 16.13276189 |  |
| Beef sausages | 24.77678664 | 16.23190541 | 23.58378482 | 16.86547291 | 22.01698921 | 11.80233335 | 13.96993284 |  |
| Ham | 4.257266585 | 2.65235888 | 3.8544188 | 3.496793875 | 4.726579887 | 1.854093589 | 3.124990804 |  |
| Potato crisps, pre-packaged | 7.022659955 | 4.741770047 | 1.928853544 | 1.787153723 | 19.15607246 | 6.068912743 | 18.9872817 |  |
| Potato chips, commercial | 16.05174473 | 9.989584904 | 5.202718404 | 4.393968109 | 17.60739772 | 4.236909002 | 5.364190753 |  |
| Ice cream | 19.18231574 | 18.28820221 | 36.95765536 | 21.94637445 | 53.28575712 | 39.95970159 | 37.78865716 |  |
| White sugar | 13.83070304 | 9.554603616 | 19.20152743 | 12.10930726 | 11.33697227 | 5.598432985 | 13.14480895 |  |
| Salad dressing | 6.446528316 | 7.035147633 | 2.596663657 | 4.631515463 | 3.190447562 | 3.124075647 | 11.07148312 |  |
| Tomato sauce | 12.26758408 | 11.7747028 | 6.753363215 | 8.655494834 | 12.19009874 | 4.400577721 | 2.952878146 |  |
| Chicken soup, canned | 44.61515881 | 46.27343161 | 72.82230414 | 54.95937126 | 0 | 4.826127294 | 18.45542827 |  |
| Orange fruit juice | 94.22031432 | 67.65290804 | 67.30679793 | 68.61274707 | 148.4931497 | 122.0261009 | 152.0821551 |  |
| Fish fillet crumbed, pre-packaged | 8.53902217 | 6.564084627 | 9.483878232 | 8.487886058 | 2.267514315 | 4.19363993 | 9.11022529 |  |
| Instant noodles, wheat based | 5.663797512 | 3.015298211 | 0.59525281 | 4.030583827 | 8.882124269 | 9.617506861 | 13.13221005 |  |
|  |  |  |  |  |  |  |  |  |
