# Supplementary material for: Healthy diets ASAP – Australian Standardised Affordability and Pricing methods protocol
Source: Nutr J. 2018 Sep 27;17:88. doi: 10.1186/s12937-018-0396-0 (PMC6161417; doi:10.1186/s12937-018-0396-0)
Supplement: Supplementary file 2 — A. Foundation diet recommended serves of foods per week for individuals (NHMRC 2011) comprising the reference household and other common households. B. Healthy (recommended) Diets: Recommended serves per day of food groups and amounts of composite foods and drinks for individuals comprising the reference household, consistent with Foundation Diets (NHMRC 2011) including commonly-consumed brands. (DOCX 55 kb) [file 12937_2018_396_MOESM2_ESM.docx]

**Additional file 2 A: Foundation diet recommended serves of foods per week for individuals (NHMRC 2011) comprising the reference household and other common households**

| **Additional file 2 B. Healthy (recommended) Diets: Recommended serves per day of food groups and amounts of composite foods and drinks for individuals comprising the reference household, consistent with Foundation Diets (NHMRC 2011) including commonly-consumed brands** | | | | | | | | | | | | | | | | | | |  |  | |  |
| --- | --- | --- | --- | --- | --- | --- | --- | --- | --- | --- | --- | --- | --- | --- | --- | --- | --- | --- | --- | --- | --- | --- |
|  | | | | | |  | | |  | |  | |  | |  | |  | |  |  | |  |
| **Food Group and Items** | | **Detailed description of food** | **Serve size** | | Adult male 19-50 yrs old | | Adult female 19-50 yrs old | Senior male 70+ yrs old | | Senior female 70+ yrs | | Boy 14 yrs old | | Girl 8 yrs old | | Boy 4 yrs old | |  | | |  |  |
|  |  | |  | | Recommended amount per day (gram or ml) | | | | | | | | | | | | | Weighted distribution of serves within group and sub-group | | |  |  |
| **FRUIT group (recommended serves per day)** | | | 150g | | 2 | | 2 | 2 | | 2 | | 2 | | 1.50 | | 1.50 | |  | | |  |  |
| Apples | | Apple, red skin, unpeeled, raw |  | | 100.00 | | 100.00 | 100.00 | | 100.00 | | 100.00 | | 90.00 | | 75.00 | | 0.33 | | |  |  |
| Bananas | | Banana, cavendish, peeled raw |  | | 100.00 | | 100.00 | 100.00 | | 100.00 | | 100.00 | | 90.00 | | 75.00 | | 0.33 | | |  |  |
| Oranges | | Orange, navel (all varieties), peeled, raw |  | | 100.00 | | 100.00 | 100.00 | | 100.00 | | 100.00 | | 90.00 | | 75.00 | | 0.33 | | |  |  |
| **VEGETABLES AND LEGUMES group (recommended serves per day)** | | | 75g | | 6 | | 5 | 5 | | 4.86 | | 5.29 | | 5.14 | | 4.29 | |  | | |  |  |
| - **Starchy veg** | |  | 75g | | 1 | | 0.71 | 0.71 | | 0.43 | | 1 | | 0.60 | | 0.50 | |  |  |  |  |  |
| - **Green and brassica** | |  | 75g | | 1 | | 1 | 1 | | 1 | | 1 | | 1.20 | | 1 | |  |  |  |  |  |
| - **Orange veg** | |  | 75g | | 1 | | 1 | 1 | | 1 | | 1 | | 1.20 | | 1 | |  |  |  |  |  |
| - **Legumes** | |  | 1/2 cup cooked 75g approx. | | 1 | | 0.29 | 0.29 | | 0.43 | | 0.29 | | 0.34 | | 0.29 | |  |  |  |  |  |
| - **Other veg** | |  | 75g | | 2 | | 2 | 2 | | 2 | | 2 | | 1.80 | | 1.50 | |  |  |  |  |  |
| Potatoes (starchy) | | Potato, coliban, peeled, boiled, microwaved or steamed, drained | 1/2 medium or 75g | | 50.00 | | 35.71 | 35.71 | | 21.43 | | 50.00 | | 30.00 | | 25.00 | | 0.66 | | |  |  |
| Canned sweet corn (starchy) | | Sweetcorn, kernels, canned in brine, drained | 1/2 cup approx. 75g | | 25.00 | | 17.86 | 17.86 | | 10.71 | | 25.00 | | 15.00 | | 12.50 | | 0.33 | | |  |  |
| Broccoli (green/brassica) | | Broccoli, fresh, boiled, microwaved or steamed, drained | 1/2 cup cooked or 75g | | 25.00 | | 25.00 | 25.00 | | 25.00 | | 25.00 | | 30.00 | | 25.00 | | 0.33 | | |  |  |
| White cabbage (green/brassica) | | Cabbage, white, boiled, microwaved or steamed, drained, with and without added fat | 1/2 cup cooked or 75g | | 25.00 | | 25.00 | 25.00 | | 25.00 | | 25.00 | | 30.00 | | 25.00 | | 0.33 | | |  |  |
| Iceberg lettuce (green/brassica) | | Lettuce, iceberg, raw | I cup or 75g | | 25.00 | | 25.00 | 25.00 | | 25.00 | | 25.00 | | 30.00 | | 25.00 | | 0.33 | | |  |  |
| Carrot (orange veg) | | Carrot, mature, peeled or unpeeled, fresh or frozen, raw | 75g | | 37.50 | | 37.50 | 37.50 | | 37.50 | | 37.50 | | 45.00 | | 37.50 | | 0.50 | | |  |  |
| Pumpkin (orange veg) | | Pumpkin, peeled, fresh or frozen, boiled, microwaved or steamed, drained | 1/2 cup cooked or 75g | | 37.50 | | 37.50 | 37.50 | | 37.50 | | 37.50 | | 45.00 | | 37.50 | | 0.50 | | |  |  |
| Canned 4 bean mix (Legumes) | | Beans, mixed, canned, drained | 1/2 cup or 75g (approx..) | | 37.50 | | 10.71 | 10.71 | | 16.07 | | 10.71 | | 12.86 | | 10.71 | | 0.50 | | |  |  |
| Canned diced tomatoes (other) | | Tomato, whole, canned in tomato juice, undrained | 1/2 cup or 75g | | 30.00 | | 30.00 | 30.00 | | 30.00 | | 30.00 | | 27.00 | | 22.50 | | 0.20 | | |  |  |
| Onion (other) | | Onion, mature, peeled, fresh or frozen, baked, roasted, fried, stir-fried, grilled or bbq'd, fat not further defined | 1 small onion or 75g | | 30.00 | | 30.00 | 30.00 | | 30.00 | | 30.00 | | 27.00 | | 22.50 | | 0.20 | | |  |  |
| Tomatoes (other) | | Tomato, common, raw | 1 medium tomato | | 30.00 | | 30.00 | 30.00 | | 30.00 | | 30.00 | | 27.00 | | 22.50 | | 0.20 | | |  |  |
| Frozen mixed vegetables (other) | | Mixed vegetables, purchased frozen, carrot, corn & pea/bean, cooked, with or without fat | 1/2 cup cooked or 75 g | | 30.00 | | 30.00 | 30.00 | | 30.00 | | 30.00 | | 27.00 | | 22.50 | | 0.20 | | |  |  |
| Frozen peas (other) | | Pea, green ,frozen, cooked, no added fat | 1/2 cup cooked or 75 g | | 30.00 | | 30.00 | 30.00 | | 30.00 | | 30.00 | | 27.00 | | 22.50 | | 0.20 | | |  |  |
| Baked beans (legumes) | | Baked beans, canned in tomato sauce, regular | 1/2 cup cooked or 75g | | 37.50 | | 10.71 | 10.71 | | 16.07 | | 10.71 | | 12.86 | | 10.71 | | 0.50 | | |  |  |
| **GRAINS (CEREAL FOODS) – Recommended serves per day** | | | 40g | | 6 | | 6 | 4.43 | | 3 | | 7 | | 4.80 | | 4 | |  | | |  |  |
| - **Wholegrain** | | | 40g bread eq | | 4 | | 4 | 3 | | 2.14 | | 4.57 | | 3.26 | | 2.71 | |  | | |  |  |
| - **Refined** | | | 40g bread eq | | 2 | | 2 | 1.43 | | 0.86 | | 2.43 | | 1.54 | | 1.29 | |  | | |  |  |
| Breakfast wheat biscuit (WG) Weetbix | | Breakfast cereal, whole wheat, biscuit, added vitamins b1,b2,b3 & folate, fe & zn | 2 wheat biscuits 40g | | 40.00 | | 40.00 | 30.00 | | 21.43 | | 45.71 | | 32.57 | | 27.14 | | 0.25 | | |  |  |
| Wholemeal bread (WG) | | Bread, from wholemeal flour ,commercial | 1 slice 40g | | 80.00 | | 68.57 | 60.00 | | 31.43 | | 91.43 | | 65.14 | | 54.29 | | 0.50 | | |  |  |
| Rolled oats (WG) | | Porridge, rolled oats, prepared with cows milk | 1/2 cup 120g | | 120.00 | | 120.00 | 90.00 | | 64.29 | | 137.14 | | 97.71 | | 81.43 | | 0.25 | | |  |  |
| White bread (refined) | | Bread, from white flour, commercial | 1 slice 40g | | 16.00 | | 16.00 | 11.43 | | 6.86 | | 19.43 | | 12.36 | | 10.29 | | 0.20 | | |  |  |
| Cornflakes Breakfast cereals (refined) | | Breakfast cereal, flakes of corn, added vitamins b1,b2,b3,c & folate, fe & zn | 2/3 cup cereal flakes 30g | | 12.00 | | 12.00 | 8.57 | | 5.14 | | 14.57 | | 9.29 | | 7.71 | | 0.20 | | |  |  |
| Pasta white (refined) | | Pasta, white wheat flour, plain, boiled from dry, no added salt | 1/2 cup cooked 75g women, older men & women_97g men & children | | 38.79 | | 30.00 | 21.43 | | 12.86 | | 47.14 | | 29.93 | | 24.93 | | 0.20 | | |  |  |
| Rice white (refined) | | Rice, white, boiled, no added salt | 1/2 cup cooked 75g women, older men & women_97g men & children | | 38.79 | | 30.00 | 21.43 | | 12.86 | | 47.14 | | 29.93 | | 24.93 | | 0.20 | | |  |  |
| Dry water cracker biscuits (refined) | | Biscuit, savoury, from white wheat flour, water cracker style | 3 crispbreads 35g | | 14.00 | | 14.00 | 10.00 | | 6.00 | | 17.00 | | 10.79 | | 9.00 | | 0.20 | | |  |  |
| sandwich from W/M bread | | Sandwich or roll, filled with chicken & salad | sandwich approx. 100g | | 11.43 | | 11.43 | 11.43 | | 11.43 | | 11.43 | | 5.71 | | 5.71 | | 0.33 each chicken, bread and salad veg | | |  |  |
| **LEAN MEATS & POULTRY, FISH, EGGS, NUTS AND SEEDS – Recommended serves per day** | | |  | |  | |  |  | |  | |  | |  | |  | |  | | |  |  |
| - **Red meat** | |  | **65g cooked** | | 1 | | 1 | 1 | | 0.43 | | 1 | | 0.86 | | 0.71 | |  | | |  |  |
| - **Non-red meat and alternatives** | | | **65 g equivalent** | | 1 | | 1 | 1 | | 1 | | 1 | | 0.93 | | 0.79 | |  | | |  |  |
| - **Nuts/seeds** | |  | **30g** | | 1 | | 0.29 | 0.57 | | 0.43 | | 0.57 | | 0.00 | | 0.00 | |  | | |  |  |
| Beef mince (red) | | Beef, mince,<5% fat, baked, roasted, fried or stir-fried ,grilled or bbq'd, no added fat | 65g | | 21.64 | | 21.64 | 21.64 | | 9.29 | | 21.64 | | 18.50 | | 15.43 | | 0.33 | | |  |  |
| Lamb chops (av fat) (red) | | Lamb, loin chop, semi-trimmed, grilled, fat not further defined | 65g | | 21.64 | | 21.64 | 21.64 | | 9.29 | | 21.64 | | 18.57 | | 15.50 | | 0.33 | | |  |  |
| Rump steak (av fat) (red) | | Beef, rump steak, semi-trimmed, grilled, fat not further defined | 65g | | 21.71 | | 21.71 | 21.71 | | 9.29 | | 21.71 | | 18.57 | | 15.50 | | 0.33 | | |  |  |
| Canned tuna in unsat oil | | Tuna, unflavoured, canned in vegetable oil, drained | 100g edible | | 33.36 | | 33.36 | 33.36 | | 33.36 | | 33.36 | | 31.43 | | 26.21 | | 0.33 | | |  |  |
| Chicken cooked | | Chicken, barbecued, with skin, commercial | 80g cooked | | 26.64 | | 26.64 | 26.64 | | 26.64 | | 26.64 | | 25.14 | | 20.93 | | 0.33 | | |  |  |
| Eggs | | Egg, chicken, whole, hard-boiled | 2 large 120g | | 40.00 | | 40.00 | 40.00 | | 40.00 | | 40.00 | | 37.71 | | 31.43 | | 0.33 | | |  |  |
| Nuts- unsalted peanuts or other nuts | | Unsalted peanuts roasted or other unsalted nuts (cheapest) | 30g | | 30.00 | | 8.57 | 17.14 | | 12.86 | | 17.14 | | 0.00 | | 0.00 | | 1.00 | | |  |  |
| **MILK, YOGHURT, CHEESE & ALTERNATIVES- Recommended serves per day** | | |  | | 2.43 | | 2.43 | 3.43 | | 4.00 | | 3.57 | | 2.00 | | 2.00 | |  | | |  |  |
| - **High fat milk (full fat cheese)** | | | **40g** | | 0.29 | | 0.29 | 0.43 | | 0.50 | | 0.43 | | 0.29 | | 0.21 | | 0.12 | | |  |  |
| - **Medium fat milk (full cream milk/yoghurt, reduced fat cheese)** | | | **250ml milk/200g yoghurt/40gcheese** | | 0.86 | | 0.86 | 1.18 | | 1.41 | | 1.27 | | 0.70 | | 0.71 | | 0.18 | | |  |  |
| - **Reduced fat milk/yoghurt** | | | **250ml milk/200g yoghurt** | | 1.29 | | 1.29 | 1.82 | | 2.09 | | 1.88 | | 1.02 | | 1.07 | | 0.70 | | |  |  |
| Hard cheddar cheese (full fat) | | Cheese, cheddar, regular fat, not further defined | 40g | | 11.43 | | 11.43 | 17.14 | | 20.00 | | 17.14 | | 10.29 | | 8.57 | | 1 high | | |  |  |
| Full cream (~4% fat) milk (medium fat) | | Milk, cow, fluid, regular fat (~3.5%),not further defined | 250ml milk | | 107.14 | | 107.14 | 147.36 | | 176.36 | | 158.50 | | 87.07 | | 89.29 | | 0.50 | | |  |  |
| Hard cheddar cheese (reduced fat)(medium fat) | | Cheese, cheddar, reduced fat, not further defined | 40g | | 8.57 | | 8.57 | 11.79 | | 14.14 | | 12.71 | | 7.00 | | 7.14 | | 0.25 | | |  |  |
| Reduced fat milk (~2% fat)/low fat milk -fresh) (reduced fat) | | Milk, cow, fluid, reduced fat (~1.5%),increased protein (~4%) | 250ml milk | | 214.29 | | 214.29 | 303.57 | | 348.29 | | 312.50 | | 116.07 | | 119.07 | | 0.66 low | | |  |  |
| Full fat plain yoghurt (<2% fat) (full fat) (medium fa) | | Yoghurt, natural, regular fat (~4%) | 200g yoghurt | | 42.86 | | 42.86 | 58.93 | | 70.57 | | 63.43 | | 34.86 | | 35.71 | | 0.25 | | |  |  |
| Reduced fat flavoured yoghurt (reduced fat) (reduced fat) | | Yoghurt, vanilla flavoured, reduced fat (~1%) |  | | 85.71 | | 85.71 | 121.43 | | 139.29 | | 125.00 | | 67.86 | | 71.43 | | 0.3 low | | |  |  |
| **Unsaturated Oils and Spreads (or foods from which these are derived): Allowance** | | | **7ml or 10 g** | | 4 | | 2 | 2 | | 2 | | 2 | | 0.86 | | 0.71 | |  | | |  |  |
| Canola margarine | | Margarine spread, monounsaturated or polyunsaturated, regular fat (>50% fat),not further defined | 10g | | 13.29 | | 6.64 | 6.64 | | 6.64 | | 6.64 | | 2.86 | | 2.36 | | 0.33 | | |  |  |
| sunflower oil | | Oil, sunflower | 7g | | 9.36 | | 4.71 | 4.71 | | 4.71 | | 4.71 | | 2.00 | | 1.64 | | 0.33 | | |  |  |
| Olive oil | | Oil, olive | 7g | | 9.36 | | 4.71 | 4.71 | | 4.71 | | 4.71 | | 2.00 | | 1.64 | | 0.33 | | |  |  |
| **Discretionary Choices- Not included in Foundation Diets for the smallest/youngest, shortest and sedentary groups** | | |  |  | |  | | |  | |  | |  | |  | |  | |  |  | |  |
