# Supplementary material for: Healthy diets ASAP – Australian Standardised Affordability and Pricing methods protocol
Source: Nutr J. 2018 Sep 27;17:88. doi: 10.1186/s12937-018-0396-0 (PMC6161417; doi:10.1186/s12937-018-0396-0)
Supplement: Supplementary file 3 — Composition of the current (unhealthy) diet and healthy (recommended) diet for four additional households (HH1, HH2, HH3, HH4)1 per fortnight. (DOC 188 kb) [file 12937_2018_396_MOESM3_ESM.doc]

**Additional file 3: Composition of the current (unhealthy) diet and healthy (recommended) diet for four additional households (HH1, HH2, HH3, HH4)[[1]](#footnote-2) per fortnight.**

| **Current (unhealthy) diet** | | | | |  | **Healthy (recommended) diet** | | | | |
| --- | --- | --- | --- | --- | --- | --- | --- | --- | --- | --- |
| **Food** | **HH1^1^** | **HH2^2^** | **HH3^3^** | **HH4^4^** |  | **Food** | **HH1^1^** | **HH2^2^** | **HH3^3^** | **HH4^4^** |
| **Total energy of basket per HH per day (kJ/day)** | **47750** | **24060** | **9800** | **14770** |  | **Total energy of basket per HH per day (kJ)** | **46400** | **24070** | **9540** | **15430** |
| **Food or drink** | **Amounts** | | | |  | **Food or drink** | **Amount** | | | |
| *Bottled water, still (ml)* | 5948 | 3275 | 2021 | 923 |  | *Bottled water, still (ml)* | 5948 | 3275 | 2021 | 923 |
| *Artificially sweetened soft drink (diet coke)* | 2660 | 1419 | 972 | 523 |  | *Artificially sweetened soft drink (diet coke)* |  |  |  |  |
| *Fruit* |  |  |  |  |  | *Fruit* |  |  |  |  |
| Apples, red, loose (g) | 5072 | 2774 | 722 | 1271 |  | Apples, red, loose (g) | 7910 | 4060 | 1400 | 2800 |
| Bananas, Cavendish, loose (g) | 1741 | 606 | 293 | 743 |  | Bananas, Cavendish, loose (g) | 7910 | 4060 | 1400 | 2800 |
| Oranges, loose (g) | 2496 | 1304 | 360 | 791 |  | Oranges, loose (g) | 7910 | 4060 | 1400 | 2800 |
| Fruit salad, canned in juice (g) | 3819 | 1425 | 621 | 2017 |  |  |  |  |  |  |
| Fruit juice | 4572 | 2367 | 3027 | 952 |  |  |  |  |  |  |
| *Vegetables* |  |  |  |  |  | *Vegetables* |  |  |  |  |
| Potato, white, loose (g) | 2181 | 944 | 516 | 1304 |  | Potato, white, loose (g) | 2970 | 1620 | 700 | 800 |
| Sweetcorn, canned, no added salt (g) | 427 | 130 | 77 | 161 |  | Sweetcorn, canned, no added salt (g) | 1485 | 810 | 350 | 400 |
| Broccoli, loose (g) | 620 | 277 | 144 | 249 |  | Broccoli, loose (g) | 2170 | 1120 | 350 | 700 |
| White cabbage, loose (g) | 331 | 141 | 94 | 174 |  | White cabbage, loose (g) | 2170 | 1120 | 350 | 700 |
| Iceberg lettuce, whole (g) | 1071 | 468 | 327 | 418 |  | Iceberg lettuce, whole (g) | 2170 | 1120 | 350 | 700 |
| Carrot, loose (g) | 1130 | 583 | 170 | 493 |  | Carrot, loose (g) | 3255 | 1680 | 525 | 1050 |
| Pumpkin (g) | 407 | 154 | 86 | 287 |  | Pumpkin (g) | 3255 | 1680 | 525 | 1050 |
| Four bean mix, canned (g) | 111 | 50 | 24 | 36 |  | Four bean mix, canned (g) | 1380 | 480 | 525 | 375 |
| Diced tomatoes, canned, in tomato juice(g) | 308 | 141 | 93 | 82 |  | Diced tomatoes, canned, in tomato juice(g) | 2373 | 1218 | 420 | 840 |
| Onion, brown, loose (g) | 124 | 37 | 48 | 128 |  | Onion, brown, loose (g) | 2373 | 1218 | 420 | 840 |
| Tomatoes, loose (g) | 712 | 301 | 187 | 423 |  | Tomatoes, loose (g) | 2373 | 1218 | 420 | 840 |
| Frozen mixed vegetables, pre-packaged (g) | 1744 | 760 | 424 | 656 |  | Frozen mixed vegetables, pre-packaged (g) | 2373 | 1218 | 420 | 840 |
| Frozen peas, pre-packaged (g) | 419 | 167 | 106 | 234 |  | Frozen peas, pre-packaged (g) | 2373 | 1218 | 420 | 840 |
| Baked beans, canned (g) | 525 | 241 | 128 | 243 |  | Baked beans, canned (g) | 1380 | 480 | 525 | 375 |
| Salad vegs in sandwich | 156 | 58 | 62 | 28 |  | Salad vegs in sandwich | 156 | 58 | 62 | 28 |
| Veg in tinned meat and vegetable casserole (g) | 872 | 432 | 214 | 312 |  |  |  |  |  |  |
| *Grain (cereal) foods* |  |  |  |  |  | *Grain (cereal) foods* |  |  |  |  |
| Wholegrain cereal biscuits Weetbix^TM^ (g) | 569 | 263 | 167 | 285 |  | Wholegrain cereal biscuits Weetbix^TM^ (g) | 2896 | 1656 | 560 | 720 |
| Wholemeal bread, pre-packaged (g) | 1680 | 683 | 370 | 1055 |  | Wholemeal bread, pre-packaged (g) | 5472 | 3152 | 1120 | 1280 |
| Rolled oats, whole (g) | 1629 | 615 | 255 | 931 |  | Rolled oats, whole (g) | 8688 | 4968 | 1680 | 2160 |
| White bread, pre-packaged (g) | 4215 | 2344 | 689 | 1093 |  | White bread, pre-packaged (g) | 1133 | 669 | 224 | 256 |
| Cornflakes (g) | 1019 | 525 | 155 | 331 |  | Cornflakes (g) | 850 | 502 | 168 | 192 |
| White pasta, spaghetti (g) | 1669 | 942 | 384 | 336 |  | White pasta, spaghetti (g) | 2571 | 1499 | 543 | 480 |
| White rice, medium grain (g) | 2207 | 1098 | 524 | 335 |  | White rice, medium grain (g) | 2571 | 1499 | 543 | 480 |
| Dry water cracker biscuit (g) | 369 | 225 | 33 | 62 |  | Dry water cracker biscuit (g) | 991 | 585 | 196 | 224 |
| Bread in sandwich | 156 | 58 | 62 | 28 |  | Bread in sandwich | 156 | 58 | 62 | 28 |
| *Meats, poultry, fish, eggs, nuts and seeds* |  |  |  |  |  | *Meats, poultry, fish, eggs, nuts and seeds* |  |  |  |  |
| Beef mince, lean (g) | 395 | 157 | 111 | 173 |  | Beef mince, lean (g) | 1514 | 865 | 303 | 433 |
| Lamb loin chops (g) | 454 | 83 | 174 | 262 |  | Lamb loin chops (g) | 1516 | 866 | 303 | 433 |
| Beef rump steak (g) | 1336 | 683 | 373 | 547 |  | Beef rump steak (g) | 1519 | 868 | 304 | 434 |
| Tuna, canned in vegetable oil (g) | 1480 | 756 | 297 | 542 |  | Tuna, canned in vegetable oil (g) | 2675 | 1374 | 467 | 934 |
| Whole barbeque chicken, cooked (g) | 2332 | 1004 | 657 | 801 |  | Whole barbeque chicken, cooked (g) | 2137 | 1098 | 373 | 746 |
| Eggs (g) | 1306 | 565 | 306 | 515 |  | Eggs (g) | 3208 | 1648 | 560 | 1120 |
| Meat in tinned meat and vegetable casserole (g) | 872 | 432 | 214 | 312 |  | Peanuts, roasted, unsalted (g) | 960 | 360 | 420 | 420 |
| Chicken in sandwich | 156 | 58 | 62 | 28 |  | Chicken in sandwich | 156 | 58 | 62 | 28 |
| *Milk, yoghurt, cheese and alternatives* |  |  |  |  |  | *Milk, yoghurt, cheese and alternatives* |  |  |  |  |
| Cheddar cheese, full fat (g) | 976 | 467 | 157 | 250 |  | Cheddar cheese, full fat (g) | 1104 | 544 | 160 | 520 |
| Cheddar cheese, reduced fat (g) | 71 | 29 | 15 | 24 |  | Cheddar cheese, reduced fat (g) | 814 | 396 | 120 | 363 |
| Milk, full fat (ml) | 8967 | 4702 | 1259 | 2027 |  | Milk, full cream (ml) | 10157 | 4938 | 1500 | 4532 |
| Milk, reduced fat (ml) | 4348 | 2102 | 828 | 2427 |  | Milk, reduced fat (ml) | 18543 | 9000 | 3000 | 9126 |
| Yoghurt, full fat plain (g) | 315 | 127 | 77 | 109 |  | Yoghurt, full fat plain (g) | 4064 | 1976 | 600 | 1813 |
| Yoghurt, reduced fat, flavoured (vanilla) (g) | 1568 | 467 | 209 | 373 |  | Yoghurt, reduced fat, flavoured (vanilla) (g) | 8050 | 3900 | 1200 | 3650 |
| Flavoured milk (ml) | 3169 | 1529 | 888 | 597 |  |  |  |  |  |  |
| *Unsaturated oils and spreads* |  |  |  |  |  | *Unsaturated oils and spreads* |  |  |  |  |
| Canola margarine (g) | 266 | 127 | 43 | 135 |  | Canola margarine (g) | 538 | 226 | 186 | 186 |
| Sunflower oil (ml) | 9 | 4 | 3 | 5 |  | Sunflower oil (ml) | 380 | 160 | 131 | 132 |
| Olive oil (ml) | 9 | 4 | 3 | 5 |  | Olive oil (ml) | 380 | 160 | 131 | 132 |
| *Discretionary choices* |  |  |  |  |  |  |  |  |  |  |
| Beer, full strength (ml) | 4905 | 428 | 4232 | 2262 |  |  |  |  |  |  |
| White wine, sparkling (ml) | 1377 | 572 | 291 | 907 |  |  |  |  |  |  |
| Whisky (ml) | 356 | 97 | 170 | 166 |  |  |  |  |  |  |
| Red wine (ml) | 1385 | 491 | 587 | 968 |  |  |  |  |  |  |
| Butter (g) | 455 | 224 | 56 | 188 |  |  |  |  |  |  |
| Muffin, commercial (g) | 2125 | 1104 | 352 | 793 |  |  |  |  |  |  |
| Cream-filled sweet biscuit, pre-packaged (g) | 807 | 359 | 137 | 410 |  |  |  |  |  |  |
| Muesli bar, pre-packaged (g) | 495 | 301 | 72 | 46 |  |  |  |  |  |  |
| Peanuts, salted (g) | 336 | 154 | 102 | 129 |  |  |  |  |  |  |
| Pizza, commercial (g) | 1448 | 872 | 310 | 169 |  |  |  |  |  |  |
| Savoury flavoured biscuits (g) | 262 | 182 | 40 | 58 |  |  |  |  |  |  |
| Confectionary (g) | 625 | 373 | 46 | 123 |  |  |  |  |  |  |
| Chocolate (g) | 528 | 333 | 108 | 109 |  |  |  |  |  |  |
| Sugar sweetened bevs (Coca Cola) (ml) | 13516 | 8612 | 3400 | 1535 |  |  |  |  |  |  |
| Meat pie, commercial (g) | 1982 | 1194 | 444 | 439 |  |  |  |  |  |  |
| Frozen lasagne, pre-packaged (g) | 5369 | 3298 | 1024 | 787 |  |  |  |  |  |  |
| Hamburger, commercial (g) | 2793 | 1749 | 664 | 352 |  |  |  |  |  |  |
| Beef sausages (g) | 1479 | 701 | 347 | 566 |  |  |  |  |  |  |
| Ham (g) | 282 | 129 | 60 | 103 |  |  |  |  |  |  |
| Potato crisps, pre-packaged (g) | 809 | 420 | 98 | 52 |  |  |  |  |  |  |
| Potato chips, hot, commercial (g) | 807 | 446 | 225 | 134 |  |  |  |  |  |  |
| Ice cream (g) | 2666 | 1561 | 269 | 825 |  |  |  |  |  |  |
| White sugar (g) | 918 | 371 | 194 | 438 |  |  |  |  |  |  |
| Salad dressing (ml) | 497 | 187 | 90 | 101 |  |  |  |  |  |  |
| Tomato sauce (ml) | 731 | 397 | 172 | 216 |  |  |  |  |  |  |
| Chicken soup, canned (g) | 2368 | 715 | 625 | 1789 |  |  |  |  |  |  |
| Orange juice (ml) | 4572 | 2367 | 3027 | 952 |  |  |  |  |  |  |
| Fish fillet crumbed, pre-packaged (g) | 548 | 182 | 120 | 252 |  |  |  |  |  |  |
| Instant noodles, wheat based (g) | 621 | 301 | 79 | 65 |  |  |  |  |  |  |

1. Abbreviation: HH Household

   HH1 (n=6): male 19-50yrs; female 19-50yrs; female 70+yrs; boy 14yrs; girl 8 yrs; boy 4 yrs

   HH2 (n=3): female 19-50yrs; boy 14 yrs; girl 8yrs

   HH3 (n=1): male 19-50 yrs

   HH4 (n=2): male 70+ yrs; female 70+yrs [↑](#footnote-ref-2)
