# Supplementary material for: Healthy diets ASAP – Australian Standardised Affordability and Pricing methods protocol
Source: Nutr J. 2018 Sep 27;17:88. doi: 10.1186/s12937-018-0396-0 (PMC6161417; doi:10.1186/s12937-018-0396-0)
Supplement: Supplementary file 5 — Median income determination by SA2 Example- Median income data from the 2011 Census, ABS Community Profiles of SA2 areas for six SA2 locations in Sydney, NSW*. (DOCX 36 kb) [file 12937_2018_396_MOESM5_ESM.docx]

|  | SA2 - NSW | Median Personal gross income (excluding govt allowances/ pensions)  ($/year) | Average household size (persons) | Median gross Household income ($/weekly)*** | Median gross Household income x1.111 ($/week)  (Includes Wage Price index increase of 11.1% from Sep 2011 – Sep 2015) | Median rent ($/week) | Median mortgage repayments ($/month) |
| --- | --- | --- | --- | --- | --- | --- | --- |
| Q1 | Lakemba-Wiley Park (119021365) (SEIFA 806-972) | $34,002 | 3.0 | $858 | $953 | $295 | $1,517 |
| Q1 | Fairfield East (125031480) SEIFA 772-963 | $37,805 | 3.1 | $824 | $915 | $225 | $1,790 |
| Q3 | Glendenning- Dean Park (116031314) SEIFA 927-1035) | $47,926 | 3.3 | $1,518 | $1,686 | $350 | $2,000 |
| Q3 | Girraween-Westmead (125041488) (SEIFA 770-1053) | $45,748 | 2.8 | $1,266 | $1,406 | $330 | $2,000 |
| Q5 | Drummoyne-Rodd Point (120011385) SEIFA 1024-1153 | $61,301 | 2.4 | $2,039 | $2,265 | $470 | $2,817 |
| Q5 | Frenchs Forest-Belrose (122031428) (SEIFA 1000-1204) | $48,403 | 3.0 | $2,037 | $2,263 | $650 | $2,900 |

*Median total household income is applicable to occupied private dwellings. It excludes households where at least one member aged 15 years and over did not state an income and households where at least one member aged 15 years and over was temporarily absent on Census Night. It excludes 'Visitors only' and 'Other non-classifiable' households.

Total Household Income (weekly) <http://www.abs.gov.au/websitedbs/censushome.nsf/home/cpexplanatorynotes?opendocument&navpos=230>

Household income is calculated by summing the individual incomes (pre-tax) reported by all household members aged 15 years and over. The Census collects individual income in ranges. Before they can be summed, a specific dollar amount needs to be allocated to each person. Median incomes for each range (derived using data from the Survey of Income and Housing) are used for this purpose.

Household income is not calculated where a household member aged 15 years and over did not state their income, or was temporarily absent. These households are coded to the Partial income stated category.

Total Personal Income (weekly) This variable indicates the total weekly income that is received and includes pensions and allowances. People are not asked to state their exact income, only to indicate the range into which their income falls. Applicable only to persons aged 15 years and over.

Median total family income is applicable to families in family households. It excludes families where at least one member aged 15 years and over did not state an income and families where at least one member aged 15 years and over was temporarily absent on Census Night.

Average household size is applicable to number of persons usually resident in occupied private dwellings. It includes partners, children, and co-tenants (in group households) who were temporarily absent on Census Night. A maximum of three temporary absentees can be counted in each household. It excludes 'Visitors only' and 'Other non-classifiable' households.

**Wage price index** is calculated quarterly. I have taken the % change over the previous year, see below, between Sep 2011 and Sep 2015 and added it to result in a 11.1% increase of wages over that period.

- Sep 2011-Sep 2012 3.7%
- Sep 2012-Sep 2013 2.6%
- Sep 203-Sep 2014 2.5%
- Sep 2014-Sep 2015 2.3%

**Limitations -** The median HH income includes all types of families and households, and does not relate to the 5 HH types of the Healthy Diets ASAP method.

**Summary of Median Incomes**

| Location | Quintile | Median Personal income (excluding govt allowances/ pensions) ($/year) | Average Household size (persons) | Median total household income ($/weekly) | Median total Household income x1.111  (To include Wage Price index increase of 11.1% from Sep 2011 – Sep 2015) | Median total Household income per fortnight^^  ($/fortnight) | Median total family income ($/weekly) | Median total family income x1.111  (To include Wage Price index increase of 11.1% from Sep 2011 – Sep 2015) ($/week) | Median rent ($/week) | Median mortgage repayments ($/month) |
| --- | --- | --- | --- | --- | --- | --- | --- | --- | --- | --- |
| NSW | Q1 | 35,904 | 3.05 | 841 | 934 | 1868 | 920 | 1022 | 260 | 1654 |
|  | Q3 | 46,837 | 3.05 | 1392 | 1547 | 3094 | 1492 | 1658 | 340 | 2000 |
|  | Q5 | 54,852 | 2.70 | 2038 | 2264 | 4528 | 2408 | 2675 | 560 | 2859 |
| ACT | Q1 | 58,073 | 2.40 | 1603 | 1781 | 3562 | 2275 | 2528 | 265 | 1985 |
|  | Q3 | 57,500 | 2.75 | 1986 | 2206 | 4412 | 2260 | 2511 | 380 | 2040 |
|  | Q5 | 70,967 | 2.75 | 2759 | 3065 | 6130 | 3114 | 3460 | 502 | 2334 |

^^Used in affordability calculations for Healthy Diets ASAP in NSW and ACT
