# Supplementary material for: Healthy diets ASAP – Australian Standardised Affordability and Pricing methods protocol
Source: Nutr J. 2018 Sep 27;17:88. doi: 10.1186/s12937-018-0396-0 (PMC6161417; doi:10.1186/s12937-018-0396-0)
Supplement: Supplementary file 6 — Calculations of low (minimum) disposable household income data from welfare data – Example. (DOCX 34 kb) [file 12937_2018_396_MOESM6_ESM.docx]

| **Additional file 6: Calculations of low (minimum) disposable household income data from welfare data - Example** | **Household 1: Household of 6** | | **Household 2: Single parent with 2 children** | | **Household 3: Single unemployed person** | | **Household 4: Older couple with no children** | | **Household 5: Two parents with two children**  **Reference Household** | |
| --- | --- | --- | --- | --- | --- | --- | --- | --- | --- | --- |
|  | Adult male, adult female, older female, 14yr boy, 8yr girl, 4yr boy | | Adult female, 14yr boy, 8yr girl | | Adult male | | Older male, older female | | Adult male, adult female, 14yr boy, 8yr girl | |
| **Assumptions** | •  The adult male and female are partnered parents of the three dependent children •  Adult male is unemployed and looking for work •  Adult female is a stay-at-home mum •  Older female receives age pension •  The older children attend school and are fully immunised •  The youngest child attends kindy 2 days/week and is fully immunised •  The family does not have savings or investments •  The family is living in public housing | Amounts per fortnight (Feb 2016) | •  The adult female works on a casual basis at national minimum wage ($21.61/hr) for 25 hours a week for 39 weeks per year (not during school holidays) •  The adult female does not receive child support from the children’s father •  Both children attend school and are fully immunised •  None of the family are disabled •  The family does not have savings or investments •  The family is privately renting their home at $376/week | Amounts per fortnight (Feb 2016) | •  Has no paid employment but is looking for work •  Is not studying/training •  Is not disabled •  Has no dependent children •  Does not have savings or investments •  Is renting a room in 3 bedroom house at $125/week ($376/3) | Amounts per fortnight (Feb 2016) | •  Neither are in paid employment •  Both receive the full age pension (maximum rate) •  Neither are disabled or frail-aged •  The couple has no dependent children •  The couple has some savings earning $100/fortnight in ‘deemed income’ (less than asset test for age pension) •  The couple is privately renting their home at $376/week | Amounts per fortnight (Feb 2016) | •  The adult male works on a permanent basis at national minimum wage for 38 hours a week ($17.29/hr) •  The adult female works on a part-time basis at national minimum wage ($17.29/hr) for 6 hours a week • Both children attend school and are fully immunised •  None of the family are disabled •  The family has some emergency savings that earn negligible interest •  The family is privately renting a 3 bedroom house at $376/week | Amounts per fortnight (Feb 2016) |
| **INCOME (fortnightly)** |  |  |  |  |  |  |  |  |  |  |
| Paid employment - adult male | Nil | - |  | - | Nil | - | Nil | - | $17.29/hr for 38h/week | $ 1,314.04 |
| Paid employment - adult female | Nil | - | $20.30/h/25h per week/39 weeks | $ 810.38 |  | - | (deemed income not included as assumed to be reinvested) | - | $17.29/hr for 6h/week | $ 207.48 |
| Newstart Allowance | $472.60/fortnight (adult male) | $ 472.60 | N/A | - | $523.40 (receives maximum) | $ 523.40 | N/A | - | N/A | - |
| Parenting Payment | $472.60/fortnight (adult female) | $ 472.60 | N/A (as youngest child is not under 8 years) | - | N/A | - | N/A | - | N/A (as youngest child is not under 8 years) | - |
| Family Tax Benefit A fortnightly payment | $593.46/fortnight | $ 593.46 | $413.70/fortnight | $ 413.70 | N/A | - | N/A | - | $413.70/fortnight | $ 413.70 |
| Family Tax Benefit A annual supplement | $726.35/child/year | $ 83.81 | $726.35/child/year | $ 55.87 |  | - |  | - | $726.35/child/year | $ 55.87 |
| Family Tax Benefit B fortnightly payment | $100.10/fortnight | $ 100.10 | $106.82/fortnight | $ 106.82 | N/A | - | N/A | - | $106.82/fortnight | $ 106.82 |
| Family Tax Benefit B annual supplement | $354.05/year/family | $ 13.62 | $354.05/year/family | $ 13.62 |  | - |  | - | $354.05/year/family | $ 13.62 |
| Age Pension fortnightly payment | $788.40 (older female) | $ 788.40 | N/A | - | N/A | - | $1,188.60 (couple payment) | $ 1,188.60 | N/A | - |
| Age Pension Fortnightly Supplement | $64.50 (older female) | $ 64.50 | N/A | - | N/A | - | $97.20 | $ 97.20 | N/A | - |
| Total Clean Energy Supplement (from all payments) | $44.18 | $ 44.18 | $9.94 | $9.94 | $8.80 | $ 8.80 | $21.20 | $ 21.20 | $9.94 | $ 9.94 |
| Rent Assistance | N/A (as live in public housing) | - | $151.76 | $ 151.76 | $86.27 | $ 86.27 | $121.80 | $ 121.80 | $151.76 | $ 151.76 |
| Income Support Bonus phased out, Sep 2016 was final payment | $92.10/biannually (adult male and female) | $ 14.17 | N/A | - | $110.60/biannually | $ 8.51 | N/A | - | N/A | - |
| Low Income Supplement | N/A | - | N/A | - | N/A | - | N/A | - | N/A | - |
| Low Income Family Supplement | N/A | - | $300/year | $ 11.54 | N/A | - | N/A | - | $300/year | $ 11.54 |
| Single Income Family Supplement | N/A | - | N/A | - | N/A | - | N/A | - | N/A | - |
| School Kid Bonus phased out, 2016 was final payment | $215/biannually for primary school child, $428/biannually for secondary school child | $ 49.46 | $215/biannually for primary school child, $428/biannually for secondary school child | $ 49.46 | N/A | - | N/A | - | $215/biannually for primary school child, $428/biannually for secondary school child | $ 49.46 |
| Childcare benefit | Paid directly to kindergarten | - | N/A | - | N/A | - | N/A | - | N/A | - |
| Childcare rebate | Paid directly to kindergarten | - | N/A | - | N/A | - | N/A | - | N/A | - |
| INCOME TAX PAID | Nil | - | Nil due to low income tax offset ($302 withheld from wages is refunded with tax return) | -$ 3.86 | Nil | - | Nil | - | $2,588/year (after allowing for low income tax offset) | -$ 99.55 |
| **FORTNIGHTLY INCOME TOTAL** |  | **$2,696.90** |  | **$ 1,619.23** |  | **$626.98** |  | **$1,428.80** |  | **$ 2,234.68** |
