# Supplementary material for: Healthy diets ASAP – Australian Standardised Affordability and Pricing methods protocol
Source: Nutr J. 2018 Sep 27;17:88. doi: 10.1186/s12937-018-0396-0 (PMC6161417; doi:10.1186/s12937-018-0396-0)
Supplement: Supplementary file 6 [file 12937_2018_396_MOESM4_ESM.docx]

Additional file 4: Energy and nutrient analysis of individual current and healthy diet baskets compared to results of the AHS and Foundation Diet modelling

Key nutrients for selected age/gender groups for non-branded foods & recommended intakes (NRVs) - Unhealthy & Healthy Diets

	Unhealthy (current) unbranded (AusNut 2013)	AHS	Healthy basket unbranded (AusNut 2013)	Recommended (NRVs)	Unhealthy (current) unbranded (AusNut 2013)	AHS	Healthy basket unbranded (AusNut 2013)	Recommended (NRVs)	Unhealthy (current) unbranded (AusNut 2013)	AHS	Healthy basket unbranded (AusNut 2013)	Recommended (NRVs)	Unhealthy (current) unbranded (AusNut 2013)	AHS	Healthy basket unbranded (AusNut 2013)	Recommended (NRVs)
**Age/Gender**	**Adult male 19-50**				**Adult female 19-50**				**Senior male 70+yo**				**Senior female 70+yo**			
**Energy kJ/day**	9,800	10,220	9,540 (1.1% dif)	Ht 1.6 PAL 1.5 9,650	7,260	7,540	7,970 (4.2% dif)	Ht 1.5 PAL 1.5 7,650	8,170	8,170	8,170 (4.7% dif)	Ht 1.6 PAL 1.5 7,800	6,600	6,570	7,260 (4.5% dif)	Ht 1.5 PAL 1.5 6,950
**% Energy from protein**	17	18	20	15-25	17	18	22	15-25	16	17	23	15-25	17	18	22	15-25
**% Energy from fat**	31	32	35	20-35	31	33	29	20-35	31	31	33	20-35	33	32	34	20-35
**% Energy from sat. fat**	13	12	10	<10	13	12	10	<10	13	12	11	<10	14	12	13	<10
**% Energy from carbohydrate**	43	43	41	45-65	44	44	44	45-65	44	45	40	45-65	43	44	39	45-65
**% Energy from alcohol**	6	6	0	<5	4	4	0	<5	5	5	0	<5	4	4	0	<5
**Protein g/day**	97	108	113	64	73	80	103	46	77	83	109	81	66	72	95	57
**Thiamin (mg/day)**	1.5	5.4	2.6	1.2	1.1	6.2		1.1	1.5	5.0		1.2	1.1	5.0		1.1
**Vitamin A as retinol equivalents (ug/day)**	776	903	1,073	900	737	903		700	911	1039		900	800	943		700
**Vitamin C (mg/day)**	80	190	108	45	62	166		45	72	148		45	63	162		45
**Folate as Dietary Folate equivalents (ug/day)**	708	778	968	400	518	672		400	697	750		400	526	640		400
**Calcium (mg/day)**	760	936	1,224	1000	617	811		1000	656	764		1300	591	783		1300
**Iodine (ug/day)**	173	209	212	150	131	172		150	162	181		150	134	158		150
**Iron (mg/day)**	12	13	17	8	9	11		18	11	12		8	8	10		8
**Magnesium (mg/day)**	293	408	416	420	222	333		320	250	328		420	206	303		320
**Zinc (mg/day)**	12	15	16	14	9	12		8	10	12		14	8	12		8

	Unhealthy (current) unbranded (AusNut 2013)	AHS	Healthy basket unbranded (AusNut 2013)	Recommended (NRVs)	Unhealthy (current) unbranded (AusNut 2013)	AHS	Healthy basket unbranded (AusNut 2013)	Recommended (NRVs)	Unhealthy (current) unbranded (AusNut 2013)	AHS	Healthy basket unbranded (AusNut 2013)	Recommended (NRVs)
**Age/Gender**	**Boy 14yo**				**Girl 8yo**				**Boy 4yo**			
**Energy kJ/day**	9,820	9,760	9,690 (2.6% dif)	PAL 1.5 9,950	6,980	6,840	6,410 (0.6% dif)	PAL 1.5 6,450	7,290	6,990	5,530 (0.4% dif)	PAL 1.5 5,550
**% Energy from protein**	15	16	22	15-25	15	15	23	15-25	14	16	23	15-25
**% Energy from fat**	33	33	29	20-35	31	31	25	20-35	33	31	26	20-35
**% Energy from sat. fat**	15	14	10	<10	14	14	9	<10	14	14	9	<10
**% Energy from carbohydrate**	49	49	45	45-65	51	53	47	45-65	50	51	46	45-65
**% Energy from alcohol**	0	0	0	<5	0	0	0	<5	0	0	0	<5
**Protein g/day**	85	92	123	65	61	61	86	20	61	65	75	20
**Thiamin (mg/day)**	1.4	2.3		1.2	1.1	2.0		0.6	1.0	2.0		0.6
**Vitamin A as retinol equivalents (ug/day)**	656	656		900	727	795		400	667	685		400
**Vitamin C (mg/day)**	85	144		40	76	116		35	85	161		35
**Folate as Dietary Folate equivalents (ug/day)**	739	735		400	576	674		200	560	655		200
**Calcium (mg/day)**	765	859		1300	672	712		700	694	811		700
**Iodine (ug/day)**	184	188		150	155	156		90	151	155		90
**Iron (mg/day)**	11	12		11	8	9		10	6	9		10
**Magnesium (mg/day)**	232	290		410	188	216		130	188	230		130
**Zinc (mg/day)**	11	12		13	8	8		4	8	8		4
